# Supplementary material for: Population sequencing enhances understanding of tea plant evolution
Source: Nat Commun. 2020 Sep 7;11:4447. doi: 10.1038/s41467-020-18228-8 (PMC7477583; doi:10.1038/s41467-020-18228-8)
Supplement: Supplementary file 1 — Supplementary Information [file 41467_2020_18228_MOESM1_ESM.pdf]

Population sequencing enhances understanding of tea plant evolution

Wang *et al.*

Supplementary Note 1. Sequencing and assembly of the Longjing 43 genome

The tea cultivar Longjing 43 (LJ43, *Camellia sinensis* var. *sinensis* cv. Longjing 43), a very popular and famous cultivar for preparing West Lake Longjing green tea in China, was selected for *de novo* assembly sequencing. LJ43 is a line selected from populations of ‘Longjing Quntizhong’, old land race populations growing in Hangzhou (TRI, CAAS, N 30°10', E 120°5') tea production areas. Since LJ43 shows excellent tea agronomic traits including early sprouting, high tea quality, and cold resistance, it has one of the largest acreages among tea plant cultivars in China, and its total cultivation area exceeds 150,000 ha in more than 10 provinces in China. Moreover, it supplies a sustainable income of more than ten billion RMB Yuan for millions of farmers per year in China.

Genomic DNA preparation and sequencing

For LJ43 Illumina shotgun library preparation, DNA was isolated from fresh leaves by 2% cetyltrimethylammonium bromide (CTAB) according to a previously published protocol[1]. A TruSeq library was prepared using the KAPA Hyper Prep Kit (Illumina® platforms, KAPA BIOSYSTEMS, Boston, MA, USA. Cat No. KK8504) following the manufacturers’ manual. All the libraries were sequenced by the HiSeq 4000 or HiSeq X® platform (Illumina®, San Diego, CA, USA) according to the manufacturer’s instructions.

Pacific Biosciences single-molecule long-read sequencing

For Pacific Biosciences (PacBio) single-molecule long-read sequencing, high-molecular-weight genomic DNA was isolated by the CTAB method[1]. A SMRTbell 25 kb needle-sheared library was constructed, size-selected with 0.375x SPRI beads, and sequenced using P6/C4 chemistry in RSII (180-min movie) according to the manufacturer's instructions. We generated 196 Gb (approximately 60-fold depth) of raw data with a read N50 of 12.5 kb and an average length of 9.1 kb.

BioNano Genomics optical mapping data generation

For BioNano Genomics optical mapping data generation, megabase-containing genomic DNA from tender shoots cultivated in the dark was prepared by a BioNano Prep™ Plant Tissue DNA Isolation Kit (BioNano Genomics, Inc., San Diego, CA, USA, Cat No. RE-014-05) according to the manufacturers' manual. The genomic DNA was fluorescently labeled using the nicking endonuclease Nt.BspQI and stained according to the manual of the IrysPrep Reagent Kit (BioNano Genomics, Inc. San Diego, CA, USA). Then, the stained DNA sample was loaded onto the nanochannel array of the IrysChip and imaged by the Irys system (BioNano Genomics, Inc. San Diego, CA, USA).

RNA isolation, RNA-seq library preparation and sequencing quality control

Total RNA from tissues of LJ43 (Supplementary Table 7) was isolated with the

RNAprep Pure Plant Kit (TIANGEN Biotech Co., Ltd., Beijing, China, Cat No. DP432), and the RNA-seq library was prepared using the KAPA RNA Hyper Prep Kit (Illumina® platforms, KAPA BIOSYSTEMS, Boston, MA, USA. Cat No. KK8541) according to the manufacturer's instructions. All the RNA-seq libraries were sequenced by the HiSeq X® platform (Illumina®, San Diego, CA, USA).

Sequencing quality control

DNA and RNA sequencing reads were trimmed and filtered using Trimmomatic (version 0.36.5)[2] after the first round of quality control using FastQC (version 0.11.5). The adapters and low-quality bases (Phred score < 20) were removed from the leading and trailing of the reads. The reads were then scanned with a 4-base-wide sliding window and cut when the average quality per base within the window dropped below 15. Reads with a length of < 75 bp were dropped, and a second round of quality control was performed using FastQC to ensure the quality of the trimmed data.

Estimation of genome size

The genome size of LJ43 was estimated by three approaches. First, using the Angiosperm DNA C-values Database (http://data.kew.org/cvalues/release 8.0, Dec 2012), the DNA C-values of *Camellia sinensis* Kuntze were estimated by Feulgen microdensitometry, and the amount of 1C DNA in was 3,824 Mb[3]. Second, an optimized DNA flow cytometry method was used for genome size estimation according to Huang's method[4,5] (Supplementary Figure 1). Third, KmerGenie

(version 1.7051)[6] was used to estimate genome size with 214 Gb of Illumina short reads. KmerGenie was run with different k-mer lengths ranging from 17 to 127 with a step size of 10. A K-mer abundance histogram was computed, and the best possible k-mer length was chosen. The predicted genome size was approximately 3.32 G with a best k-mer length of 97 (Supplementary Figure 2).

LJ43 genome assembly

Approximately 196 Gb (approximately 60-fold depth) of PacBio reads was used for LJ43 genome assembly with WTDBG (version 1.2.8). WTDBG was run with the parameters (-fo dbg --load-alignments dbg.alignments --edge-min 3 --rescue-low-cov-edges). The assembled genome was corrected with PacBio reads and approximately 214 Gb (approximately 66-fold depth) of Illumina PE 150 reads. First, PacBio reads were used to correct the genome by Arrow (version 2.1.0) with default parameters**.** Then, Illumina short reads were mapped to the previous step-corrected genome by bwa (version 0.7.15)[7] with default parameters, variants were called using BCFtools (version 1.6), and all of the homozygous mutation sites were removed by an in-house-developed script. Polishing stopped when the number of corrected bases reached a plateau. After 7 rounds of Illumina read correction, a 3.2 Gb tea genome with a contig number of 37,600, contig N50 of 271.33 kb, and GC content of 38.67% was obtained. We compared the genome with the published Yunkang10 (YK10) and Shuchazao (SCZ) genomes (Table 1).

Hi-C library preparation and sequencing

The leaves of LJ43 were treated with formaldehyde to fix nuclear chromatin. The fixed chromatin was digested with the MboI enzyme. The free blunt ends were ligated by biotinylated nucleotides. Then, the DNA was purified, and the fragments with biotinylated nucleotides were extracted. Sequencing libraries were generated according to the manufacturer's instructions (Illumina). After PCR enrichment, three libraries were sequenced and produced 263 Gb of clean PE150 data.

Hi-C-assisted genome assembly

The 10x reads were first processed by Long Ranger v2.2.2 to create an interleaved file of barcoded pair-end reads. Then, we aligned the barcoded paired-end reads to the genome with BWA-MEM (version 0.7.15) with the '-pC' parameter. The alignment file was processed using the ARCS (version 1.0.6) + LINKS (version 1.8.6) pipeline to create a Graphviz Dot file (.gv) with contig head/tail length for masking alignments set to 50 kb. LINKS was then used to join nodes in the graph produced by ARCS with default parameters[8,9]. Both the contig versions obtained by the PacBio assembly and the scaffold version obtained by the PacBio assembly with 10x scaffolding were used as input for Hi-C scaffolding. Clean paired-end reads were aligned to the genome using BWA (version 0.7.15). Then, the mapping results were filtered with a mapping quality ≥20 and edit distance (NM) ≤5. We also filtered the alignment file to only keep reads aligned to the region within 500 bp around a restriction site. The final alignment file was fed to Lachesis. To obtain the final set of

Lachesis parameters, we randomly varied the parameters through 10,000 scaffolding iterations. These randomized parameter sweeps varied within the following bounds: CLUSTER_MIN_RE_SITES, between 1 and 5,000; CLUSTER_MAX_LINK_DENSITY, between 1 and 30; ORDER_MIN_N_RES_IN_TRUNK, between 1 and 5000; and ORDER_MIN_N_RES_IN_SHREDS, between 1 and 5000. The result for the contig version of the genome showed a fraction of sequences in orderings with high orientation quality: 17,715 (76.47%), with a length of 2,801,038,355 bp (93.56%). The result for the scaffold version of the genome showed a fraction of sequences in orderings with high orientation quality: 12,288 (59.31%), with a length of 2,840,497,749 bp (92.13%). The interaction heatmap of the scaffold version showed more errors than that of the contig version (Supplementary Figure 3 and 4). Moreover, we compared collinear protein blocks with *Actinidia chinensis* using MCScanX. The contig version also showed better results (3,205 genes in blocks of the contig version vs 3,053 in those of the scaffold version), indicating that incorrectly joined scaffolds were probably caused by 10x data. Considering this, we finally used the contig version for Hi-C anchoring. After the preliminary test above, Annoroad Gene Technology performed further polishing due to limited computing resources.

The final Hi-C-assisted genome assembly was commissioned by Annoroad Gene Technology. Approximately 1,266,516,127 clean paired-end reads were used to improve the LJ43 genome assembled by the PacBio reads using HiC-Pro (version

2.7.8)[10]. First, the reads were mapped to the genome (PacBio assembly genome) by Bowtie2. Then, the results were filtered by extracting the unique mapped paired-end reads. HiC-Pro was used to locate the unique paired-end reads mapped to contigs. Lachesis[11] was used to scaffold the contigs into 15 chromatin clusters by agglomerative hierarchical clustering (Supplementary Table 2 and 3; Figure 1b; Supplementary Figure 5). A total of 7,071 contigs consisting of 2,311,549,792 bp (70.9%) were ordered with orientation. The resulting scaffold N50 was 143,847,529 bp.

Evaluation of LJ43 genome assembly quality

A total of 49,529 ESTs of tea were downloaded from the NCBI and mapped to the tree tea genome by GMAP (2017-10-30)[12]. We filtered the results according to a coverage ≥ 90% and an identity ≥ 90%. A total of 35,240 (approximately 71.15%) ESTs were mapped to LJ43, 35,202 (approximately 71.07%) ESTs were mapped to SCZ, and 31,303 (approximately 63.20%) ESTs were mapped to YK10. The results showed that the LJ43 genome was more complete than previously published tea genomes.

We evaluated completeness at the genome level by Benchmarking Universal Single-Copy Orthologs (BUSCOs) and found that the completeness of the LJ43 genome was 90.3%, with 3.2% fragments and 6.5% missing. Afterwards, we also checked whether the completeness of gene annotation was caused by incomplete

assembly of the genome. The missing positions of fragmented genes were extracted from the BUSCO alignment results and compared to the annotation file. If the fragmented genes were due to contig breakage, the missing parts would be located at either end of the contig. We found that 24 (out of 90) fragmented genes in the LJ43 genome were due to contig breakage, which was due to the complexity and heterozygosity of the tea genome. Although we attempted to increase the number of complete genes, contig breakage resulted in 24 fragmented genes.

Supplementary Note 2. Genome annotation

Repeat sequences and Transposable Elements

Repeat sequences were identified by combining *de novo* annotation and homology-based methods. For *de novo* repeat sequence prediction, RepeatModeler (1.0.4, http://www.repeatmasker.org/RepeatModeler.html) was used to search for repetitive sequences in the genome, and then the results were used to build a repeat sequence library. After that, RepeatMasker (v. 2.1, http://www.repeatmasker.org) was applied to identify repeat sequences by the repeat sequence library (Supplementary Table 5). For homology-based prediction, the genome assembly was compared to Repbase of RepeatMasker and RepeatProteinMask. Then, the predicted transposable elements (TEs) were combined by removing redundant TEs. TE repeat annotation was revealed to be up to ~2.30 Gb and comprised approximately 70.44% of the tea genome (Supplementary Table 6).

We used LTR-finder (version 1.05)[13] to search the LJ43 genome, and 35,380 intact LTR retrotransposons were obtained. Then, the 5' and 3' LTR sequences were aligned with Muscle (version 3.8.31)[14], and the Kimura two-parameter distance was calculated using EMBOSS (version 6.4.0) for each intact LTR. The insertion time between varieties was calculated according to the formula Time=Ks/2μ ($\mu = 6.5\times10^{-9}$ mutations per site per year). The SCZ and YK10 genomes were analyzed in the same way. Comparison of the results of the three tea genomes showed that the LJ43 genome had more recently inserted LTR retrotransposons (Supplementary Figure 6). We compared the PacBio read-corrected genome and NGS read-corrected genome to verify that the different 5' and 3' terminal IR sequences of LTR were real and not caused by NGS read correction (Supplementary Figure 6d). The presence of more recent LTR retrotransposons in the LJ43 genome indicated that it is more complete than the SCZ and YK10 genomes. LTR-retriever[15] was used to identify long repeat retrotransposons, and then LAI[16] was used to evaluate the LTR assembly index.

Protein-coding gene prediction

The protein-coding genes were annotated by combining *ab initio* prediction and homology-based prediction. To facilitate protein-coding gene prediction, we generated a total of approximately 340 Gb of RNA-seq clean data from 19 samples collected from 5 tissues (bud, leaf, flower, stem, and root) in four seasons (except for flowers during summer) and three biological replicates for each sample (Supplementary Table 7). First, we used PASA (version 2.0.0)[17] to build a

comprehensive transcriptome library. Then, unigenes with CDS lengths longer than 900 bp and an all vs all identity less than 70% were selected to train Augustus (version 3.3)[18] and GlimmerHMM (version 3.0.4)[19]. Afterwards, Augustus and GlimmerHMM with the default parameters were trained by the selected unigenes for *ab initio* prediction. For the homology-based predictions, we used the homologous proteins annotated in the genomes of Arabidopsis[20], rice[21], coffee[22], coca[23], and grape[24]. First, GenblastA[25] was used to cluster the adjacent HSPs (high-scoring pairs) from the same protein alignments, and GeneWise (version 2.4.1)[26] was used to identify accurate gene structures. Then, clean RNA-seq reads were mapped to the LJ43 genome by TopHat2[27]. Subsequently, Cufflinks (version 2.2.1) was used to predict gene models. All of the above results were integrated with EVidenceModeler (version 1.1.1)[28], and protein-coding genes with both CDS lengths shorter than 300 nt and stop codons were filtered (except those with a stop codon at the end of the sequence). Then, RNA-seq reads were mapped against the predicted coding regions by Soap2[29], and the predicted gene regions were selected by RNA-seq data (coverage >50%). Finally, a total of 33,556 genes supported by transcription reads were identified in the annotation. To calculate the transcript-level expression, RNA-seq reads were mapped to the genome by HISAT2 (version 2.1.0) with default parameters, and transcript-level expression was analyzed by StringTie (version 1.3.3b) and Ballgown with default parameters[30].

The average length of the LJ43 genome (10,816 kb) was longer than that of the

shuchazao[31] (SCZ, 7,386 kb) and Yunkang 10[32] (YK10, 3,549 kb) genomes. The completeness of the gene set for LJ43 was higher than that for SCZ and YK10, which may explain why the average gene length of LJ43 was longer than that of SCZ and YK10. We also compared the gene length and gene length distribution of LJ43 to those of six other species (SCZ[31], YK10[32], citrus[33], *Amborella trichopoda*[34], *Actinidia chinensis*[35], and *Gingko biloba*[36]). The average gene lengths of LJ43, SCZ, YK10, citrus, *Amborella trichopoda*, *Actinidia chinensis*, and *Gingko biloba* were 10,816 bp, 7,386 bp, 3,549 bp, 4,061 bp, 11,053 bp, 5,388 bp, and 25,619 bp, respectively. LJ43 and SCZ had more genes with lengths between 10 and 50 kb, and LJ43, *Amborella trichopoda* and *Ginkgo biloba* had more genes with lengths ≥ 50 kb (Supplementary Figure 7). The average length for LJ43 was similar to that for *Amborella trichopoda*.

If the long gene among our gene annotations was reliable, the exon-exon junction in the longest and shortest gene set should have a similar ratio supported by RNA-seq. Thus, we selected genes with high expression (FPKM values ≥ 20) in any tissue or period with more than two exons (14,698 genes). Among the selected genes, the 1,000 longest genes contained 11,446 exon-exon junctions, and 4,796 were supported by RNA-seq reads. The 1,000 shortest genes contained 1,270 exon-exon junctions, and 631 were supported by transcription reads. The supported and unsupported exon-exon junctions of the longest genes and shortest genes were tested by the Chi-square test in R, and the *P*-value was 0.5345, indicating that there was no significant difference in reliability between the longest genes and shortest genes. Therefore, the gene

annotation of LJ43 was reliable.

Evaluation of the genome annotation

For further quantitative assessment of annotation completeness, the three genome annotations were evaluated by BUSCO[37] with the same default parameters. Embryophyta Ortholog Database 10 (embryophyta_odb10, https://busco.ezlab.org/datasets/prerelease/embryophyta_odb10.tar.gz) was used. LJ43, SCZ and YK10 had 88.4%, 80.6% and 68.6% complete genes, respectively (Table 1). The detailed results for LJ43 were as follows: 88.4% complete (single: 80.9%, duplication: 7.5%), 6.9% fragmented, and 4.7% missing. The results for SCZ were 80.6% complete (single: 73.3%, duplication: 7.3%], 9.5% fragmented, and 9.9% missing. The results for YK10 were 68.6% complete (single: 64.2%, duplication: 4.4%), 16.7% fragmented, and 14.7% missing.

Homology search and functional annotation of the LJ43 genome

All of the predicted genes were functionally annotated according to homologous alignments with BLASTP (e-value ≤1e-5) against the Swiss-Prot and TrEMBL databases. InterProScan (version 5.21)[38] was further used to predict gene ontologies (GO terms) and domain information. The Kyoto Encyclopedia of Genes and Genomes (KEGG) automatic annotation server (KAAS) was used to assign putative gene functions to KEGG pathways. Using homologous alignments and domain scanning integrated with pathway annotation, 93.69% (31,437) of the protein-coding genes had

significant similarities in functional protein databases; among them, 78.59% (26,373), 91.35% (30,655), 59.71% (20,035), and 25.76% (8,643) could be assigned functions by the SwissProt, InterPro, GO, and KEGG databases, respectively (Supplementary Table 8).

Supplementary Note 3. Comparative genomic analysis

Syntonic block analysis

The protein sequences of LJ43 and *Actinidia chinensis*[35] were analyzed by blastp with the parameters -evalue 1e-5 -num_alignments 5. Then, syntenic blocks were identified by MCScanX[39] with the parameters –e 1e-20. SCZ and YK10 were analyzed with the same pipeline and parameters. The genome synteny of *Theobroma cacao*[23] with LJ43, SCZ and YK10 was also analyzed. Compared with that of *Actinidia chinensis*[35], the genomes of LJ43, SCZ and YK10 contained 690, 111 and 54 colinear blocks, respectively. A total of 18,030, 1,487, and 393 genes were involved in the above collinear blocks, respectively. Compared to *Theobroma cacao* L., LJ43 had 413 colinear blocks with 14,661 genes; SCZ had 233 colinear blocks with 3,047 genes; and YK10 had 0 colinear blocks with 0 genes.

Construction of a phylogenetic tree and estimation of gain and loss of gene families

To characterize the gene families that experienced gene gain and loss in the tea

genome, a phylogenetic tree was constructed for LJ43, *Actinidia chinensis*[35], *Coffea*[22], *Theobroma cacao*[23], *Arabidopsis thaliana*[20], *Oryza sativa* subsp. *japonica*[21,40], *Populus trichocarpa*[41], *Amborella trichopoda*, and *Vitis vinifera*[24]. A total of 1,031 single-copy gene families were identified among 9 genomes. The longest alternatively spliced genes were chosen to reconstruct the phylogeny. OrthoMCL[42] was used to cluster the gene families. The coding sequences of the single-copy genes were concatenated to a supergene sequence for each species. The supergenes were aligned by MAFFT[43]. The aligned sequences were used for phylogenetic analyses by raxml-HPC-MPI-AVX[44]. *Amborella trichopoda* was chosen as the outgroup in our analysis. The output of OrthoMCL and phylogenic tree structure were used for computational analysis of changes in gene family size with the software CAFE.

The expanded families were UDP-glucuronosyl/UDP-glucosyltransferase (GO:0016758, *P*-value < 2.20E-16, FDR < 2.40E-14), which catalyzes glucosyl transfer in flavanone metabolism and is related to catechin content; (-)-germacrene D synthase (K15803, *P*-value = 8.01E-06, FDR = 0.91E-03), which catalyzes the conversion of farneyl-PP to germacrene D and is related to terpene metabolism; NB-ARC (GO:0043531, *P*-value < 2.20E-16, FDR < 2.40E-14), Bet v I/Major latex protein (GO:0009607, *P*-value = 4.49E-04, FDR = 8.64E-03), RPM1 (K13457, *P*-value < 2.20E-16, FDR < 1.25E-13) and RPS2 (K13459, *P*-value = 8.88E-08, FDR = 2.51E-05), which are related to disease resistance; and the S-locus glycoprotein domain (GO:0048544, *P*-value < 2.20E-16, FDR < 2.40E-14), which is associated with self-incompatibility.

Positively selected genes

It has previously been reported that overexpression of cationic peroxidase 3 (OCP3)[45] (Cha14g001590) and Serpin-ZX[46] (Cha09g003010) is involved in disease resistance, whereas that of beta-glucosidase-like SFR2 (SFR2, Cha05g001710) is involved in freezing tolerance[47]. Other identified genes include one involved in the maintenance of photosystem II under high-light conditions (MPH1[48], ChaUn21494.1) and a photosystem II 22-kDa protein (PSBS, Cha09g008070) that protects plants against photooxidative damage.

Whole-genome duplication of LJ43 and the diversity of three teas

We selected SCZ, YK10 and 9 additional species as mentioned above to build gene families by OrthoMCL. To estimate the divergence time of the LJ43 paralogs, we selected gene families consisting of exactly 2 tea genes to calculate the Ks of the pairs. We obtained 3,233 2-member gene clusters for tea. Yn00 of PAML[49] was used to calculate the ks value. The peak of the Ks distribution of gene pairs was approximately 0.31. The divergence time was calculated according to the formula Time=Ks/2μ and was based on a molecular clock (μ) with a substitution rate of 6.1×10^{-9} mutations per site per year for eudicots[50].

MCScanX was used to detect the syntenic genes of LJ43 and SCZ. We selected orthologous genes to calculate Ks by Yn00 of PAML. This approach was also used for YK10. The peak of the Ks distribution of the LJ43 and SCZ gene pairs was

approximately 0.003. The peak of the Ks distribution of the LJ43 and YK10 gene pairs was approximately 0.045 (Supplementary Figure 11).

Supplementary Note 4. Sequencing and analysis of the tea populations

Samples, DNA extraction and sequencing to obtain population data

The 139 tea accessions occupying a wide range of the species distribution were collected worldwide, including 105 from East Asia, 7 from South Asia, 9 from Southeast Asia, 6 from western Asia, 7 from Africa, and 5 from Hawaii (Figure 2a, Supplementary Data 3). The average sequencing depth was approximately 13-fold (Supplementary Data 3).

DNA was isolated from fresh leaves by 2% CTAB according to a previously published protocol[1]. The TruSeq library was prepared using the KAPA Hyper Prep Kit (Illumina platforms, Kappa Biosystems, Boston, USA, Cat No. KK8504) according to the instructions of the manufacturer. DNA from each sample was randomly fragmented by nebulization to an average size of 400 bp and processed by the Illumina DNA sample preparation protocol, including end-repair, tail-adding, paired-end adaptor ligation and PCR. Paired-end sequencing libraries of each sample were built with an insert size of 400 bp, and sequencing was performed on a HiSeq 2000 platform with a read length of 150 bp.

Among the identified single nucleotide polymorphisms (SNPs), a total of 188,323,429 (89.37%) were located in intergenic regions, followed by 20,506,072 (9.73%) in introns and 1,756,690 (0.834%) in CDS regions. For the SNPs in CDS regions, 995,686 (0.47%) were missense variants and may have a strong effect on related gene function. Furthermore, 18,186 (54.20%) annotated genes were affected in at least one accession. In addition, 734,138 (0.35%) synonymous SNPs occurred in CDS regions (Supplementary Table 15).

Admixture of tea populations

To further illustrate the evolutionary history of the tea genome, a model-based clustering algorithm implemented in Admixture was used to estimate the relative genome composition for each accession. The clustering algorithm analysis indicated that the three populations fit the best model for all 139 accessions (Supplementary Figure 12). When k was 3, *C. sinensis* var. *sinensis* (CSS), *C. sinensis* var. *assamica* (Masters) Chang (CSA), and *C. sinensis*-related species (CSR) could be distinguished; this was consistent with the principal component analysis (PCA) result (Figure 2d). When k was 3 or 4, most of the new accessions collected from China appeared to have originated from CSA and CSS (yellow color, marked with an arrow), indicating their high diversity.

Historical effective population size

Contigs longer than 100 kb were analyzed using thee Multiple Sequentially

Markovian Coalescent (MSMC)[51] approach to infer the historical effective population size from multiple individuals of the same population. BEAGLE (version 4.1) was used to phase the genotype calls, as the MSMC method is better suited for phased data. The phased VCF file was filtered according to a base quality greater than 20 and mapping quality greater than 30, and the depth was between 1/2 and twice the mean depth. This mask file was generated via maskBed.pipeline.sh in MSMC-tools. The mappability mask file was generated via the pipeline documented in maize. Four/six samples from each group with a high sequencing depth were selected, and each sample was treated as a haploid. We also used MSMC to assess the timing and nature of population separation.

High heterozygosity maintains tea plant adaptability

Tea has self-incompatibility and high heterozygosity. We wanted to identify the regions that tend to maintain heterozygosity in tea and the advantages of high heterozygosity. We examined the high-heterozygosity and high-deviation-ratio regions by calculating the heterozygosity and deviation ratio in a sliding window of 20 kb by steps of 2 kb. The deviation ratio was calculated by (Ho-He)/He (Ho: average observed heterozygosity, He: average expected heterozygosity). We collected the intersection of top 1% of deviation ratio regions and top 1% of heterozygosity regions.. We obtained 655 genes in the region. The genes that may maintain tea heterozygosity were collected (Supplementary Table 20). Genes related to disease resistance, growth and development, self-incompatibility, terpene synthase, and

flavanone metabolism were highly heterozygous and had a high deviation ratio. These genes may be closely related to the adaptability of tea. We further examined the high-heterozygosity points in CDS regions and found that half of the genes had high-heterozygosity points in these CDS regions, and approximately 2/3 of those points could result in nonsynonymous mutations.

Gene flow in tea

To test whether admixture confounds the phylogeny, the population allele frequency-based model in TreeMix was applied to account for variance arising from secondary migration events. The groups were split by the phylogenetic tree (Supplementary Data 9). When up to six migration events were included in the model, the major branching patterns in our tree remained largely unchanged. The results showed high admixture among the populations (Supplementary Figure 16). The results for some accessions in the phylogenetic tree were inconsistent with traditional classification. An F4 test and F3 test of the accessions were performed by TreeMix (Supplementary Table 21). When the Z-score was greater than 3 or less than -3, the results of the F4 test indicated that there was gene flow between the samples[52]. Gene flow affected the HZ114, HZ104, HZ122, and HZ050 positions in the phylogenetic tree.

We also randomly generated 1000 groups for the F4 test. Every group contained three randomly selected individuals and CM-1 (outgroup). Gene flow was detected in 979 groups. This result showed extensive gene flow among tea accessions. The

detailed results are provided in the attachment (Supplementary Data 10).

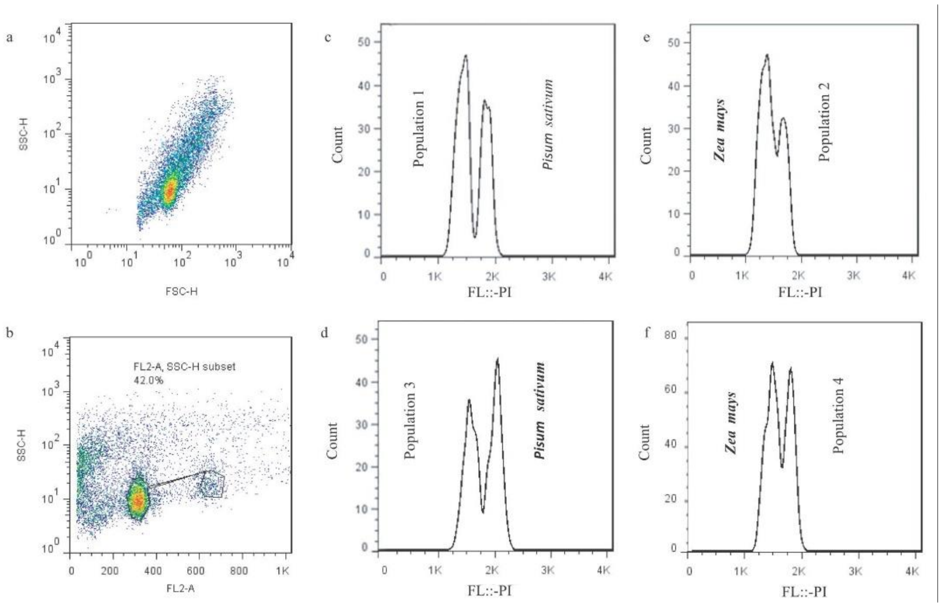

Supplementary Figure 1. Evaluation of the genome size of LJ43 by flow cytometry. a, Gating strategy for the flow cytometric histogram for measurement of genome size of four populations of tea plants. The forward scatter-side scatter (FSC-SSC) plot shows the size of the nuclei of the two plants. b, The side scatter versus FL2A channel shows the presence of florescence-labeled nuclei. Gating was performed on this panel to reduce the background signal. c-f, Four populations of LJ43 were used to determine the genome size. Two populations were estimated with *Pisum sativum* as the internal standard, and the other were estimated with *Zea mays* as the internal standard. Population 1, population 3 and population 4 contained 3 repeat leaf samples, and population 2 contained 4 repeat leaf samples. The overall mean was 3295.397087 Mb ± 179.5980113 Mb. Source data are provided as a Source Data file.

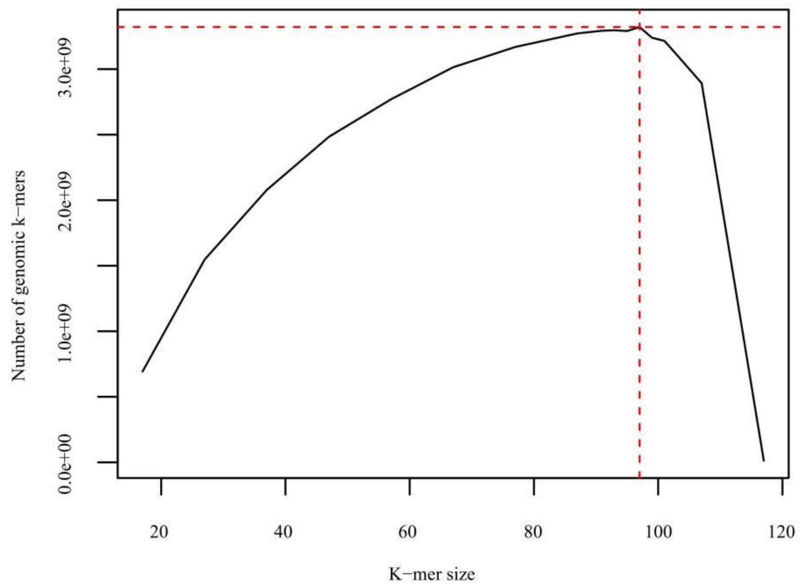

Supplementary Figure 2. The genome size of LJ43 estimated by K-mer analysis. The best k was 97 according to KmerGenie. The predicted genome size was approximately 3,321,109,494 bp.

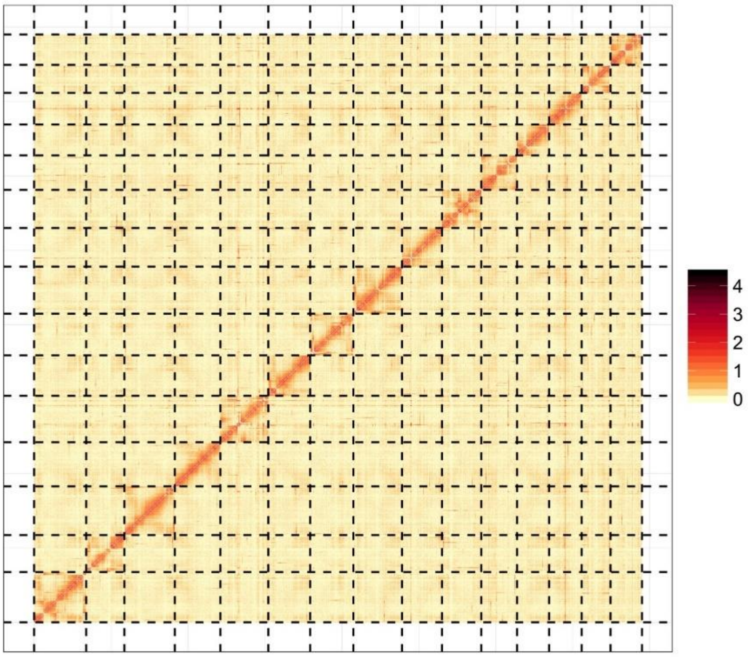

Supplementary Figure 3. Genome-wide all-by-all Hi-C interactions of the scaffold Hi-C genome. The fraction of contigs in orderings with high orientation quality was 12288 (59.31%) with a length of 2840497749 bp (92.13%). The scaffold genome has 205 collinear blocks containing 3,053 genes.

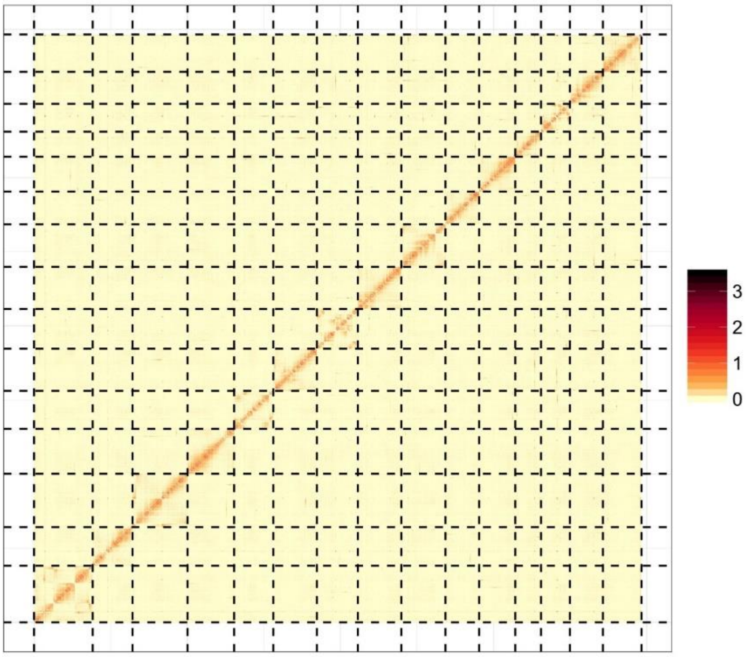

Supplementary Figure 4. Genome-wide all-by-all Hi-C interactions of the contig Hi-C genome. The fraction of contigs in orderings with high orientation quality was 17715 (76.47%), with a length of 2801038355 bp (93.56%). The contig Hi-C genome comprises 208 collinear blocks containing 3,205 genes.

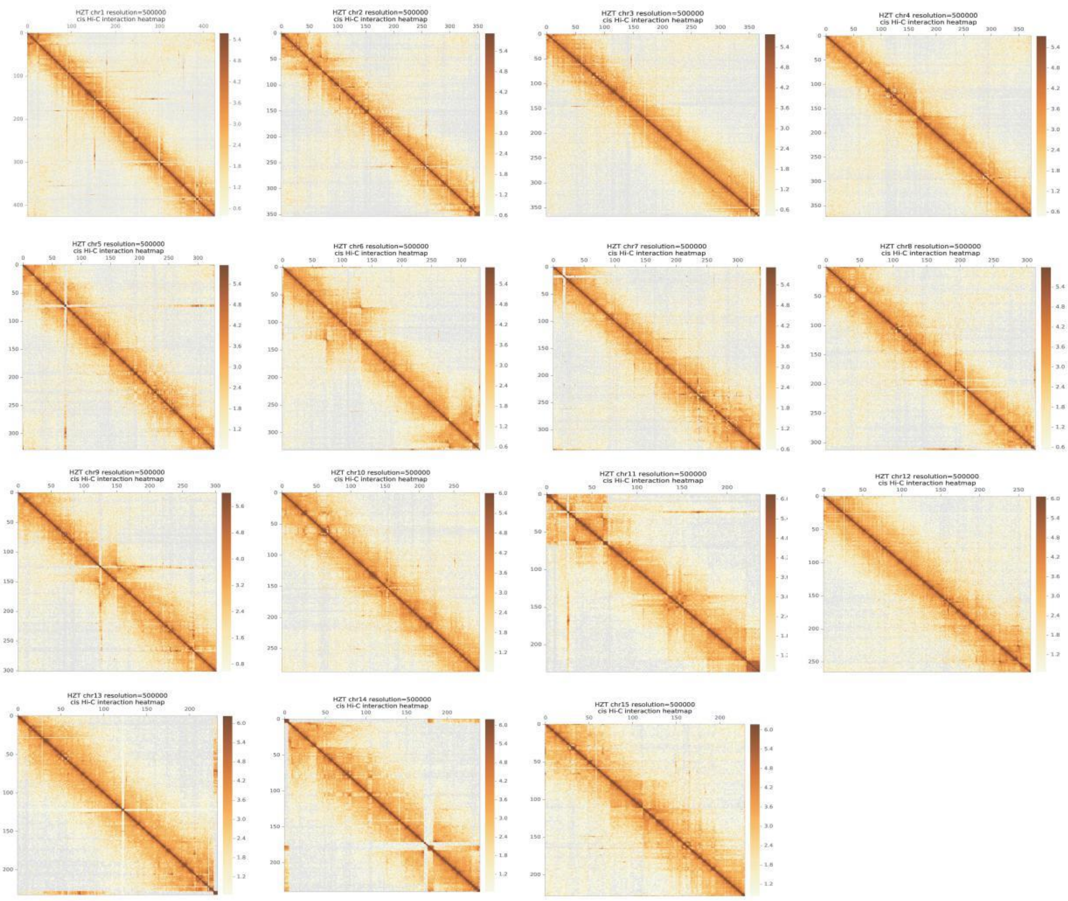

Supplementary Figure 5. Hi-C interaction within chromosomes. The resolution is 500 kp.

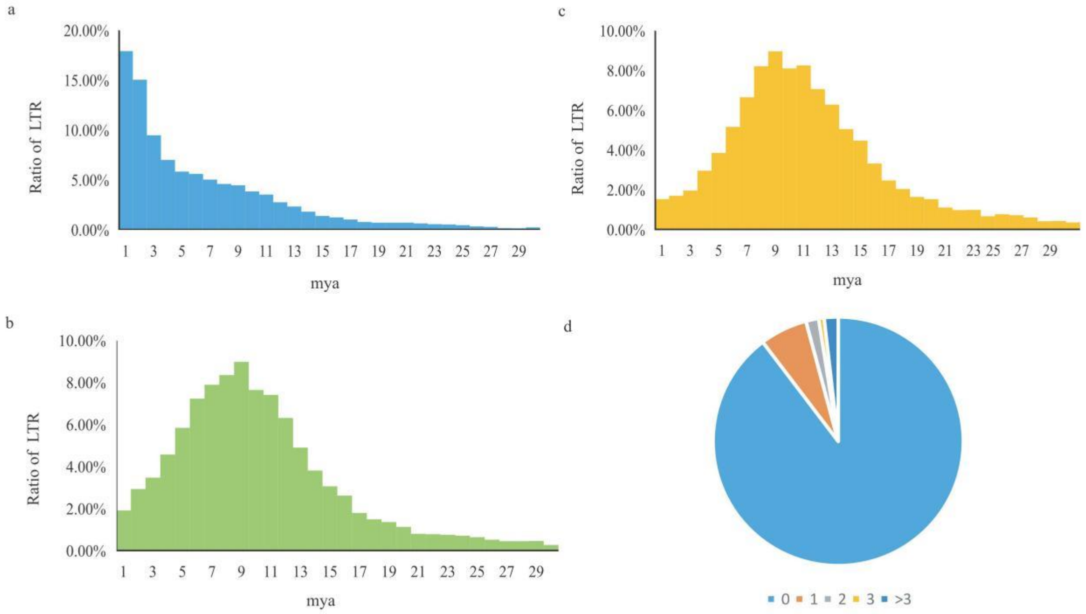

Supplementary Figure 6. The insertion times of annotated LTRs in the three tea genomes and the number of corrected bases of the LTR terminal sequence in 'Longjing 43'. a, b, and c are the insertion times of the LTRs in the genomes of LJ43, SCZ, and YK10, respectively. The abscissa is million years ago (mya), and the ordinate is the percentage of LTRs. d. The number of bases corrected by Illumina reads in complete LTR terminal sequences. The number of corrected bases of most LTR terminal sequences was 0, and the percentage of corrected bases (≤3) was approximately 98.19%. The results show that the recent LTRs in LJ43 were true and not introduced by error correction. The genome of LJ43 had more recent LTRs, implying that its genome is more complete. Source data are provided as a Source Data file.

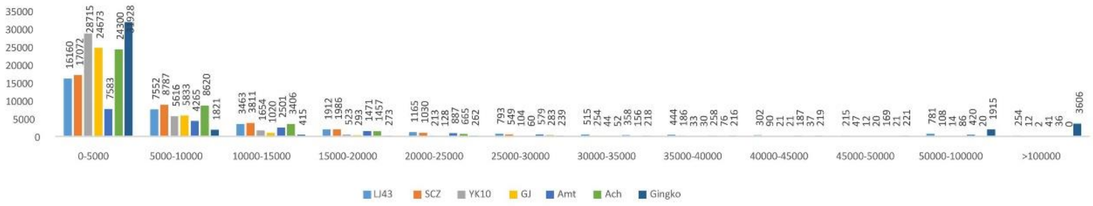

Supplementary Figure 7. The gene length distribution of seven species. LJ43, SCZ, YK10, GJ, Amt, Ach, and Gingko are Longjing 43, Shuchazao, Yunkang 10, citrus[33], *Amborella trichopoda*, *Actinidia chinensis*, and *Gingko biloba*, respectively. The X-axis shows the gene length, and the y axis shows the gene number. LJ43 and SCZ had more genes with lengths of 10 kb-50 kb, and LJ43, Amt and ginkgo had more genes with lengths ≥ 50 kb. The average gene lengths of LJ43, SCZ, YK10, GJ, Amt, Ach, and Gingko were 10,816 bp, 7,386 bp, 3,549 bp, 4,061 bp, 11,053 bp, 5,388 bp, and 25,619 bp, respectively. Source data are provided as a Source Data file.

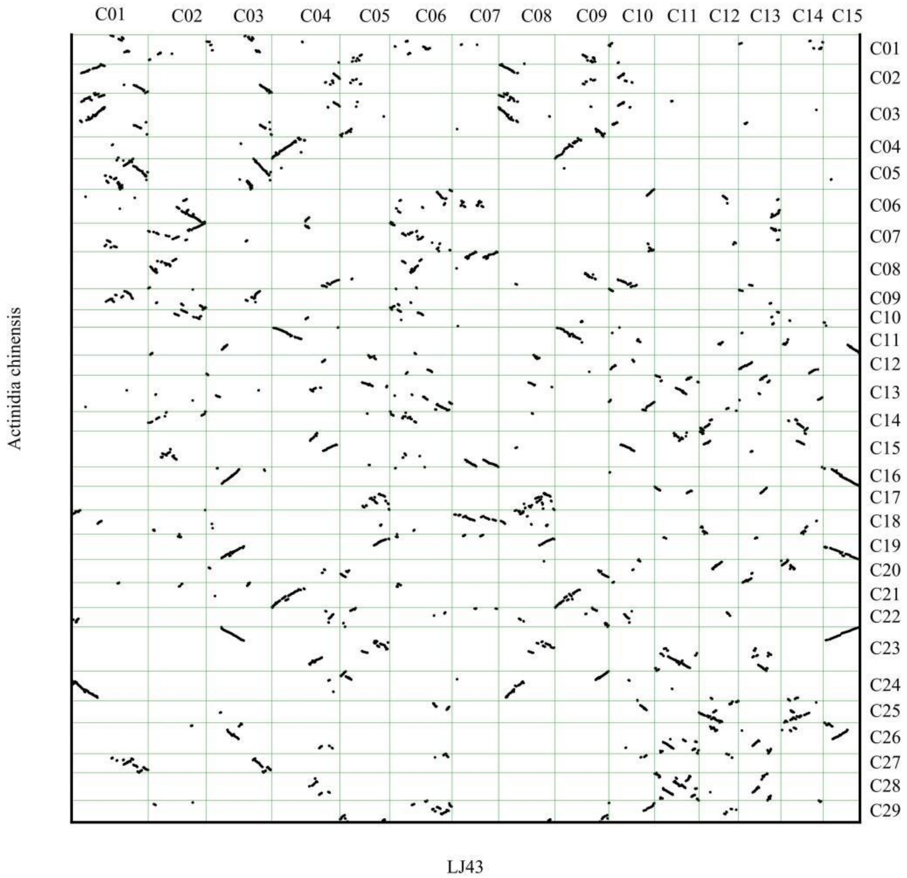

Supplementary Figure 8. The collinearity of *Actinidia chinensis* and LJ43. The chromosome-level *Actinidia chinensis* genome assembly (y axis) aligned to the chromosome-level LJ43 genome assembly (x axis). C is the chromosome. Source data are provided as a Source Data file.

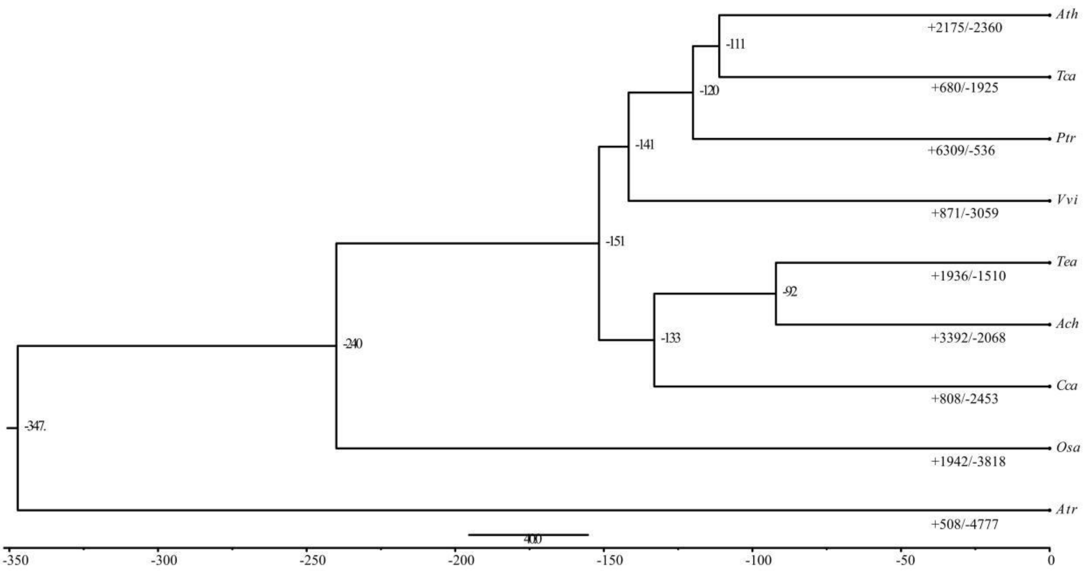

Supplementary Figure 9. Expansion and contraction of gene families in LJ43 and 8 other plant species. The divergence time is shown beside each node, and the unit is million years. Ath, Tca, Ptr, Vvi, Tea, Ach, Cca, Osa, and Atr represent *Arabidopsis thaliana, Theobroma cacao, Populus trichocarpa, Vitis vinifera,* LJ43*, Actinidia chinensis, Coffea, Oryza sativa* subsp. *geng* and *Amborella trichopoda*, respectively. *Amborella trichopoda* was the outgroup. '+' indicates expansion of gene families, and '-' indicates contraction of gene families.

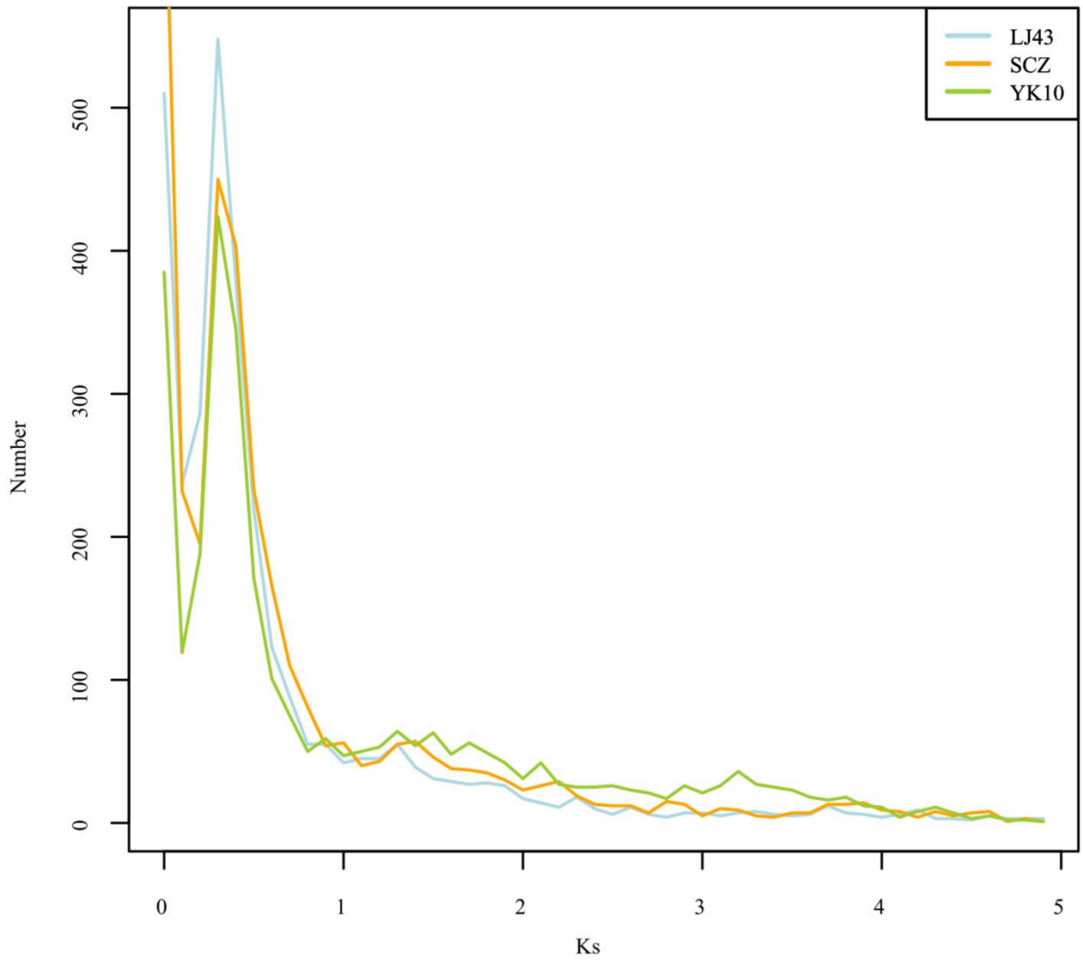

Supplementary Figure 10. Whole-genome duplication in LJ43, SCZ and YK10. The gene pairs were selected from 2-member gene groups. The x axis is Ks. The y axis is the number of gene clusters with this degree of divergence. Source data are provided as a Source Data file.

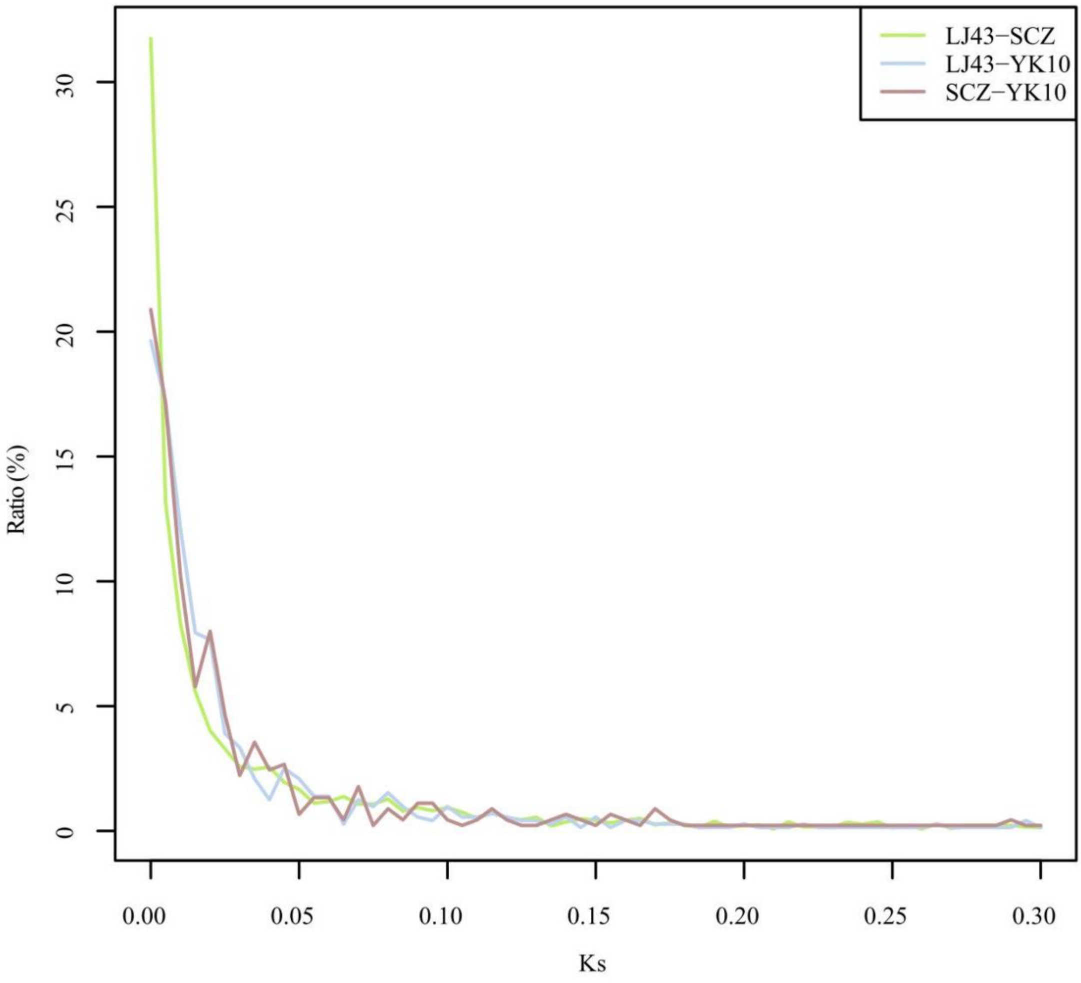

Supplementary Figure 11. The diversity of LJ43, SCZ and YK10. The x axis is the Ks of collinear genes in two tea genomes. The y axis is the number of gene pairs with this degree of divergence. Source data are provided as a Source Data file.

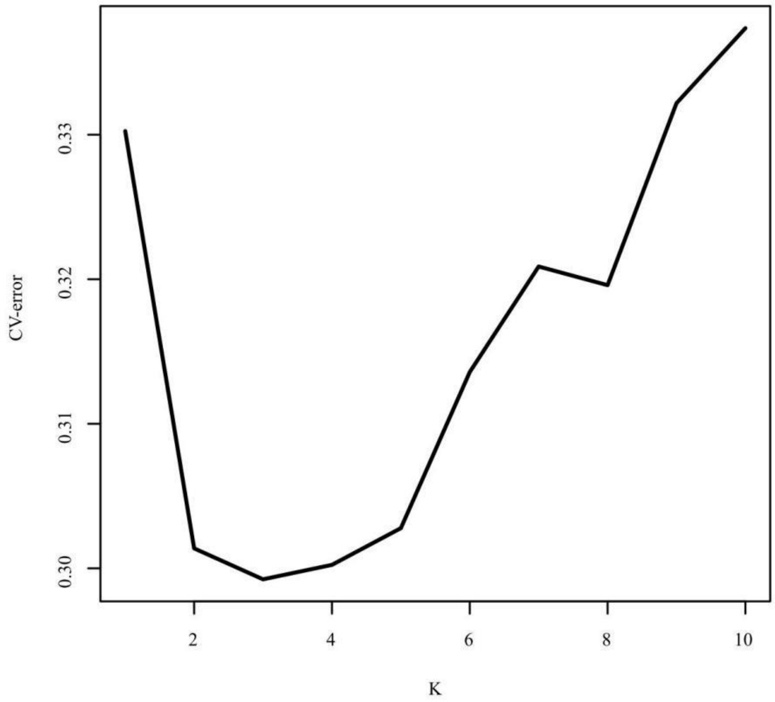

Supplementary Figure 12. The CV error in the tea populations. The CV error was calculated by Admixture, and k=3 had the lowest CV error. Source data are provided as a Source Data file.

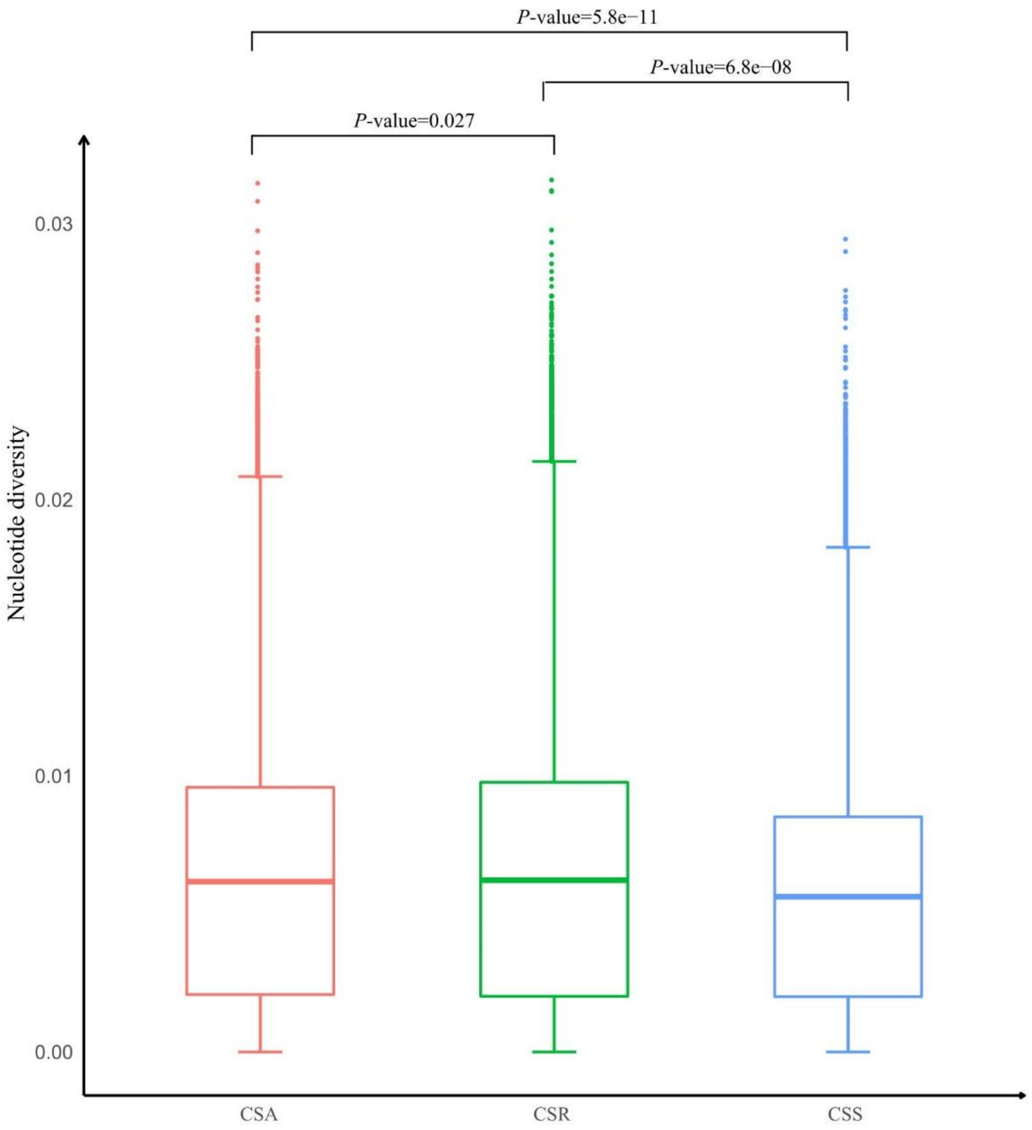

Supplementary Figure 13. The nucleotide diversity of CSA, CSR, and CSS. The boxes from left to right are CSA, CSR, and CSS. The *P*-values of all were less than 0.05. The nucleotide diversity was calculated by a 50 kb window with a step size of 10 kb, and CSA (n=42), CSR (n=10), and CSS (n=87) contained 331828, 330513, and 332162 windows, respectively. The two-sided Wilcoxon test was used to test for significance. Source data are provided as a Source Data file.

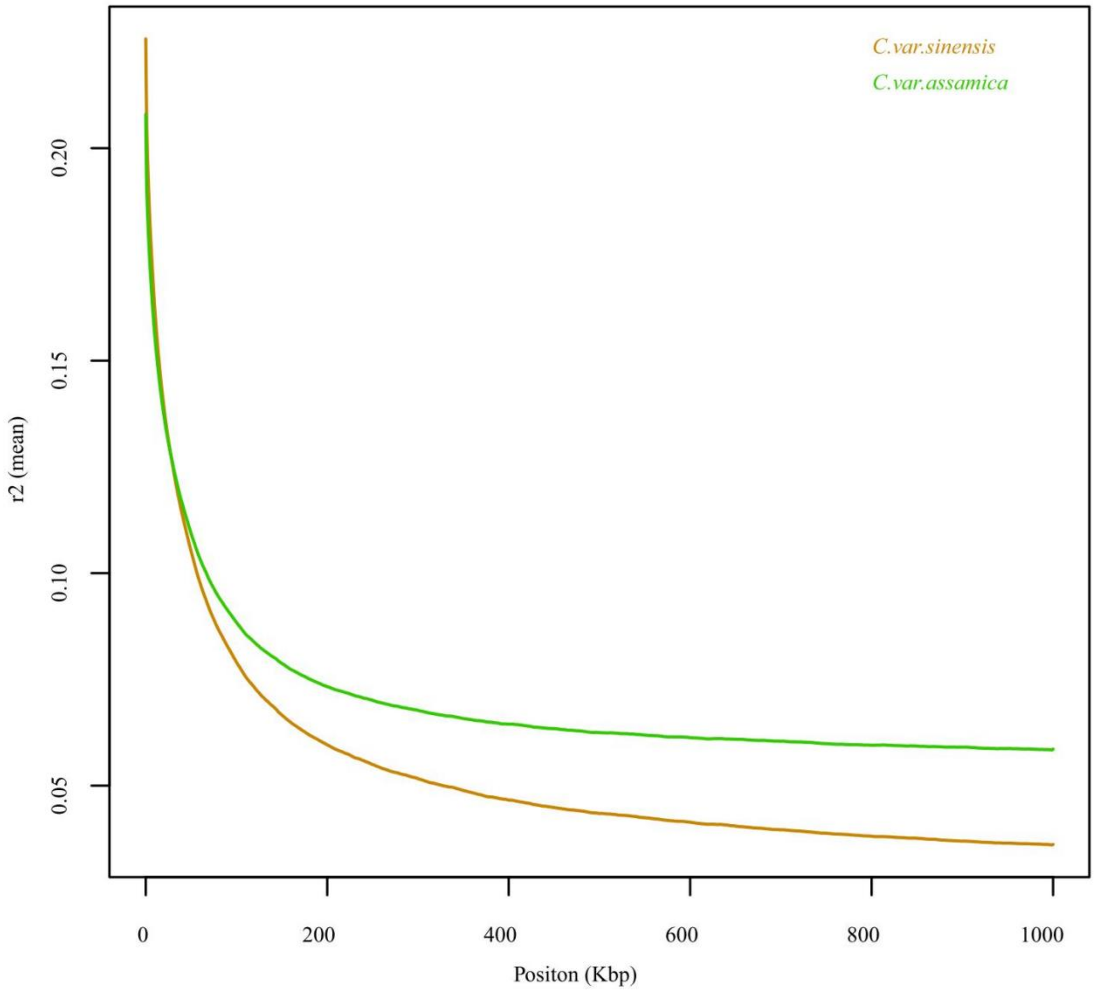

Supplementary Figure 14. LD in the tea population. Squared correlation coefficient (r^2) of linkage disequilibrium (LD) between the SNPs called in CSS and CSA populations. The average r^2 among SNPs decayed to approximately 50% of its maximum value at approximately 41 kb and 59 kb in CSS and CSA, respectively. These values indicate that the tea genomes have relatively long LD distances and slow LD decay. Source data are provided as a Source Data file.

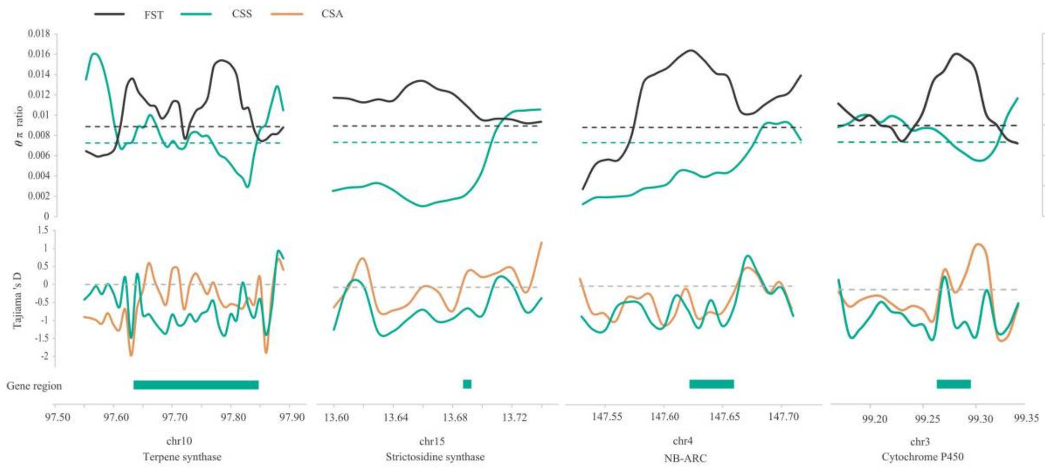

Supplementary Figure 15. The Fst, θπ ratio and Tajima's D of some selected genes. The region of selected genes had a high *Fst* value, low θ_π value and below-zero Tajima’s D. Source data are provided as a Source Data file.

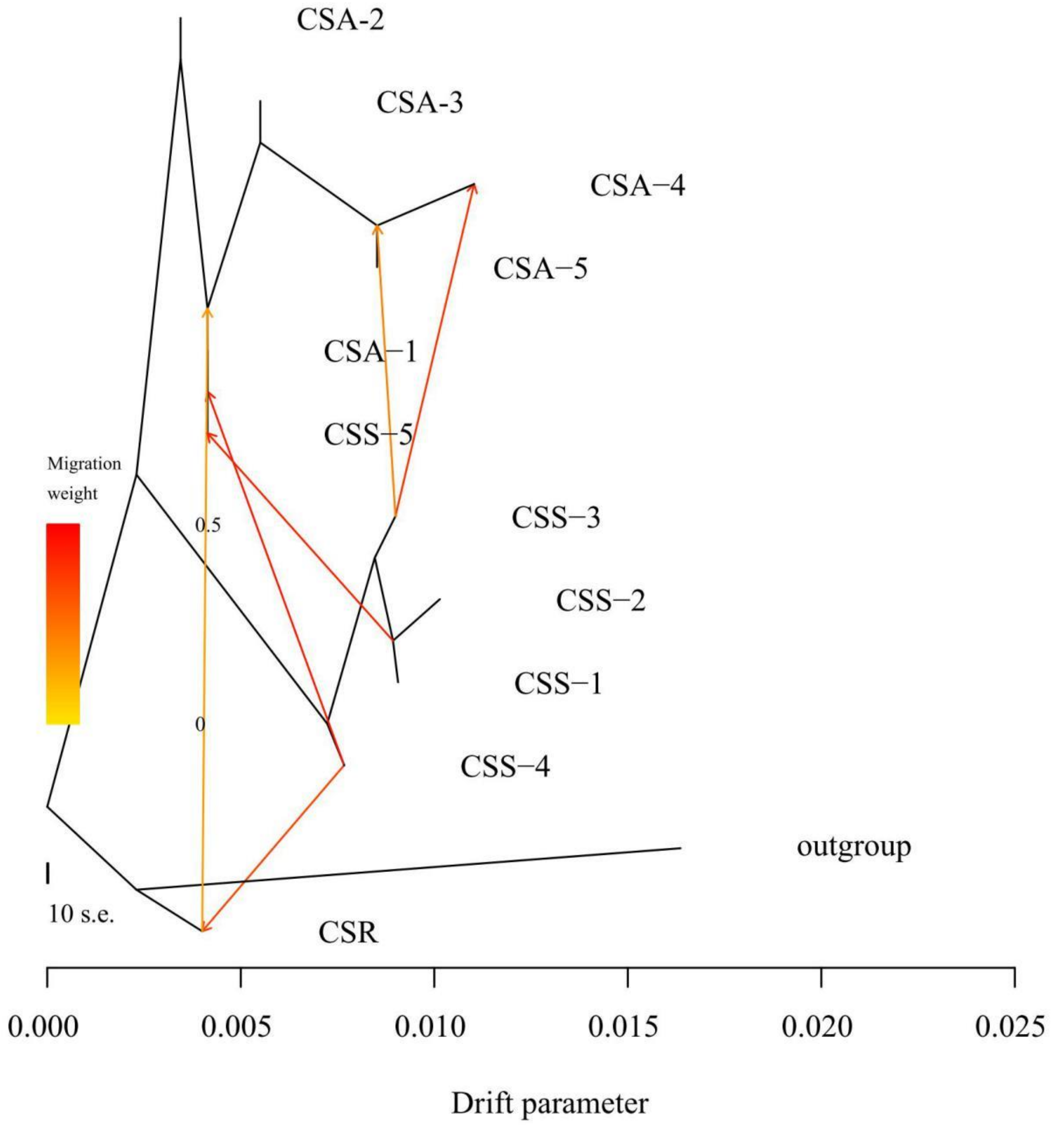

Supplementary Figure 16. The gene flow among the tea populations. The direction of each arrow represents the direction of gene flow. Information on the groups is provided in Supplementary Table 27. Source data are provided as a Source Data file.

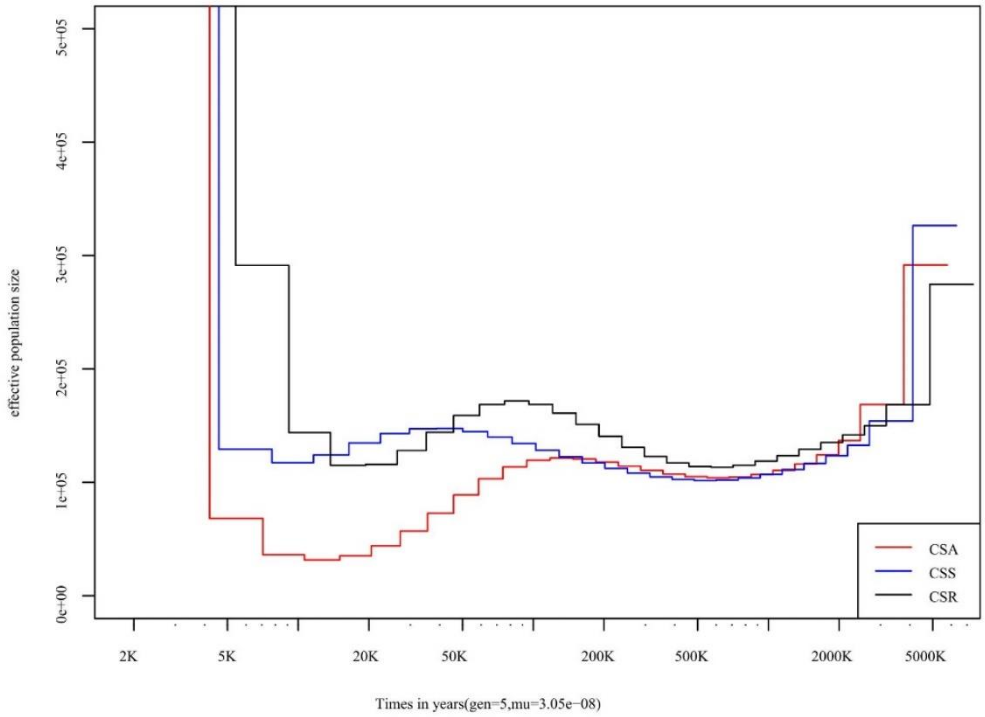

Supplementary Figure 17. The history of the tea populations. The generation time is 5 years. K is 1000 years. The change in CSR with respect to the other groups occurred at a point where CSA and CSS were very similar, which may provide additional evidence to suggest that the origins of CSA and CSS occurred at similar time points. Source data are provided as a Source Data file.

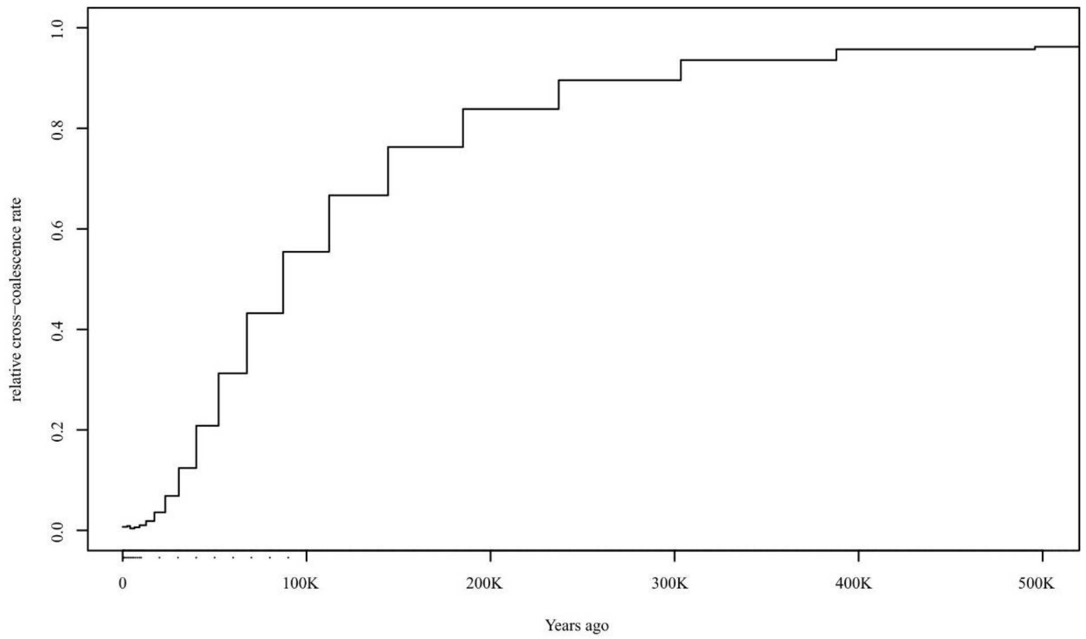

Supplementary Figure 18. The relative cross-coalescence rate of CSA and CSS. K is 1000 years. Source data are provided as a Source Data file.

Supplementary Table 1. Statistics of sequence data.

Library	Clean Data
PE	~214 Gb
PacBio	~196 Gb
RNA-seq	~340 Gb
Hic (PE150)	~263 Gb
10X (PE150)	~247 Gb
BioNano	~445 Gb

Supplementary Table 2. State of the tea genome.

Items	Contig_len (bp)	Scaffold_len (bp)	Contig_num	Scaffold_num
Total	3,259,965,435	3,260,671,035	37,600	30,544
Max_length	2,426,329	212,836,541	-	-
Number>=2000 bp	-	-	37,600	30,544
N50	271,332	143,847,529	3,214	10
N60	195,993	120,058,857	4,625	12
N70	134,207	114,499,148	6,629	15
N80	80,686	81,145	9,749	2,695
N90	35,684	35,688	15,729	8,671

Note: ‘-’ indicates missing data.

Supplementary Table 3. State of chromosome length and coefficient of determination.

Pseudomolecule	Scaffold Number	Length (bp)	Coefficient of Determination (R^2)
chr1	593	212,836,541	0.96
chr2	614	176,915,850	0.96
chr3	568	183,890,167	0.94
chr4	545	186,373,698	0.90
chr5	529	164,367,897	0.89
chr6	498	165,692,707	0.97
chr7	517	168,674,417	0.88
chr8	476	156,400,541	0.98
chr9	456	150,683,510	0.98
chr10	453	143,847,529	0.91
chr11	401	118,392,857	0.92
chr12	401	132,670,986	0.85
chr13	348	116,245,087	0.96
chr14	366	120,058,857	0.98
chr15	306	114,499,148	0.84
Total anchored	7,071	2,311,549,792	-
Unanchored	30,529	949,121,243	-

Note: ‘-’ indicates missing data.

Supplementary Table 4. The variations and InDels in LJ43.

Type	Number	Ratio
Variations (heterozygous)	19,753,302	0.606%
Variations (homogeneous)	7,307	0.000224%
InDel (heterozygous)	2,264,855	0.0695%
InDel (homogeneous)	18,525	0.000568%

Supplementary Table 5. Repetitive sequence annotation from RepeatMasker.

Elements	Number	Length (bp)
DNA_elements	342,783	166,374,592
ERV_classI	5,144	3,432,108
ERV_classII	474	344,906
hAT-Charlie	340	97,911
L3/CR1	5,031	1,160,068
LINE1	73,377	57,624,329
LINE2	9,844	3,445,887
LINEs	121,399	76,115,524
Low_complexity	123,740	6,408,906
LTR_elements	767,684	1,417,058,775
Satellites	11,812	32,810,223
Simple_repeats	869,486	105,405,729
SINEs	16,385	3,174,787
Small_RNA	17,992	5,066,448
Total_interspersed_repeats		2,448,318,482
bases_masked		2,381,432,965

Supplementary Table 6. The TEs in LJ43.

	Repbase TEs		TE proteins		*De novo*		Combined TEs	
Type	Length (Bp)	% in genome	Length (Bp)	% in genome	Length (Bp)	% in genome	Length (Bp)	% in genome
DNA TE	20,381,582	0.63	13,417,865	0.41	197,436,270	6.06	209,854,625	6.44
LINE	634,240	0.02	21,127,314	0.65	94,769,667	2.91	101,288,844	3.11
SINE	115,772	0.00	0	0.00	4,014,840	0.12	4,057,783	0.12
LTR-retro	366,724,615	11.25	464,352,782	14.24	1,964,618,648	60.27	1,981,304,145	60.77
Total	387,854,727	11.90	497,370,701	15.26	2,260,839,425	69.36	2,302,505,397	70.44

Supplementary Table 7. Transcriptome sequence of LJ43.

Name	Tissue type	Clean Reads repeat 1	Clean data (bp) repeat 1	Clean Reads repeat 2	Clean data (bp) repeat 2	Clean Reads repeat 3	Clean data (bp) repeat 3
leaf summer	mature leaves	204,015,168	7,509,164,019	185,432,168	6,848,866,161	196,054,632	7,225,952,895
leaf autumn	mature leaves	134,536,224	4,960,492,402	148,311,944	5,345,086,650	199,661,960	7,312,697,492
leaf winter	mature leaves	133,755,424	4,816,136,977	135,592,000	4,890,266,334	142,236,600	5,124,935,707
leaf spring	mature leaves	172,916,408	6,344,736,952	212,148,920	7,786,169,626	215,747,576	7,929,395,621
root autumn	roots	164,994,272	5,980,998,613	143,619,888	5,211,047,770	200,609,576	7,321,219,607
root summer	roots	150,369,688	5,490,083,176	126,524,824	4,620,519,432	159,517,416	5,818,447,162
root winter	roots	118,735,432	4,273,133,810	146,046,888	5,255,500,404	132,702,536	4,782,931,542
root spring	roots	155,342,192	5,660,781,661	129,662,080	4,757,721,164	130,815,552	4,806,046,193
flower winter	flowers	154,929,968	5,579,880,513	146,720,784	5,289,249,128	125,384,736	4,524,026,055
flower autumn	flowers	115,593,368	4,152,920,821	154,444,920	5,547,858,358	191,818,400	7,003,668,404
flower (young fruit) spring	young fruit	217,866,304	8,002,440,801	159,085,104	5,844,261,629	185,071,736	6,776,869,697
stem autumn	young stems	136,883,416	4,929,267,997	128,878,040	4,636,834,266	198,809,720	7,251,424,376
stem summer	young stems	192,130,136	7,065,984,704	184,804,800	6,796,434,130	195,752,824	7,197,948,142
stem winter	young stems	127,212,360	4,586,059,072	161,788,424	5,821,583,681	152,565,648	5,620,530,317
stem spring	young stems	132,921,200	4,880,375,054	111,220,840	4,086,690,304	127,033,040	4,664,850,247
bud autumn	axillary buds	192,373,512	7,093,878,955	197,798,048	7,283,645,328	175,308,432	6,451,941,331
bud winter	axillary buds	147,912,040	5,324,502,387	115,592,528	4,167,683,576	134,121,152	4,826,824,784
bud summer	axillary buds	234,540,448	8,622,286,106	210,273,200	7,733,356,033	185,376,328	6,842,916,444
bud spring	one bud and two leaves	177,138,808	6,495,895,305	143,576,968	5,184,808,153	263,048,184	9,662,721,977

Supplementary Table 8. Gene function annotation for LJ43.

Database	Number (ratio)
SwissProt	26,373 (78.59%)
InterPro	30,655 (91.35%)
KEGG	8,643 (25.76%)
GO	20,035 (59.71%)
Combined total annotated	31,437 (93.69%)
Unannotated	3,502 (6.31%)

Supplementary Table 9. GO enrichment of expanded genes.

GO	All Genes	Expanded Genes	*P*-value	FDR	Function
GO:0006376	4	4	2.83E-03	4.41E-02	Luc7-related
GO:0006421	4	4	2.83E-03	4.41E-02	Asparagine-tRNA ligase
GO:0008146	39	25	5.33E-08	2.18E-06	Sulfotransferase domain
GO:0008234	66	31	1.71E-05	5.09E-04	Ulp1 protease family, C-terminal catalytic domain
GO:0009607	28	15	4.49E-04	8.64E-03	Bet v I/Major latex protein
GO:0016758	311	176	2.20E-16	2.40E-14	UDP-glucuronosyl/UDP-glucosyltransferase
GO:0016891	7	6	8.46E-04	1.54E-02	Dicer dimerization domain
GO:0016998	25	14	0.38E-03	7.77E-03	Glycoside hydrolase, family 19, catalytic
GO:0030246	177	67	6.80E-06	2.47E-04	Galactose mutarotase-like domain
GO:0030247	73	42	2.44E-10	1.33E-08	Wall-associated receptor kinase, galacturonan-binding domain
GO:0031683	8	7	2.22E-04	4.84E-03	Guanine nucleotide binding protein (G-protein), alpha subunit
GO:0042545	61	27	2.05E-04	4.78E-03	Pectinesterase, catalytic
GO:0043531	436	268	2.20E-16	2.40E-14	NB-ARC
GO:0045735	60	29	1.60E-05	5.09E-04	Cupin 1
GO:0046488	19	13	3.35E-05	9.14E-04	Phosphatidylinositol-4-phosphate 5-kinase, core
GO:0048268	15	11	5.20E-05	1.31E-03	Phosphoinositide-binding clathrin adaptor, domain 2
GO:0048544	152	123	2.20E-16	2.40E-14	S-locus glycoprotein domain
GO:0051740	4	4	0.28E-02	0.44E-01	Ethylene receptor
GO:0055085	770	245	1.18E-08	5.52E-07	ABC transporter type 1, transmembrane domain
GO:0070588	23	23	2.19E-15	1.79E-13	P-type ATPase, subfamily IIB
GO:0071805	34	26	6.55E-11	4.28E-09	Potassium transporter

Note: One-sided Fisher's exact test was used to test for significance.

Supplementary Table 10. KEGG enrichment of expanded genes.

KEGG	All genes	Expanded genes	*P*-value	FDR	Function
K01183	34	18	1.49E-04	0.71E-02	chitinase
K05391	14	11	1.75E-05	0.17E-02	CNGC; cyclic nucleotide gated channel, plant
K06617	7	7	3.47E-05	0.22E-02	raffinose synthase [EC:2.4.1.82]
K07437	7	7	3.47E-05	0.22E-02	CYP26A; cytochrome P450 family 26 subfamily A
K08237	9	9	1.85E-06	0.26E-03	Glycosyltransferase Metabolism
K11835	8	7	0.22E-03	0.92E-02	Ubiquitin system Genetic Information Processing
K11844	7	6	0.85E-03	0.03	Ubiquitin system Genetic Information Processing
K13260	6	6	0.15E-03	0.71E-02	CYP81E1_7; isoflavone/4'-methoxyisoflavone 2'-hydroxylase
K13457	56	43	2.20E-16	1.25E-13	disease resistance protein RPM1
K13459	16	14	8.88E-08	2.51E-05	disease resistance protein RPS2
K13691	11	9	6.38E-05	0.36E-02	Glycosyltransferases Metabolism
K15095	10	8	2.28E-04	0.92E-02	(+)-neomenthol dehydrogenase
K15639	10	10	4.26E-07	8.03E-05	CYP734A1, BAS1; PHYB activation tagged suppressor 1 [EC:1.14.-.-]
K15803	8	8	8.01E-06	0.91E-03	GERD; (-)-germacrene D synthase
K18819	7	7	3.47E-05	0.22E-02	GOLS; inositol 3-alpha-galactosyltransferase [EC:2.4.1.123]

Note: One-sided Fisher's exact test was used to test for significance.

Supplementary Table 11. IPR enrichment of special groups in LJ43.

IPR	All genes	Special Genes	*P*-value	FDR	Function
IPR000163	13	5	0.19E-02	0.49E-01	Prohibitin
IPR002182	436	52	0.81E-03	0.32E-01	NB-ARC
IPR004045	86	17	0.22E-03	0.13E-01	Glutathione S-transferase, N-terminal
IPR008218	7	5	4.60E-05	0.36E-02	ATPase, V1 complex, subunit F
IPR009600	4	3	0.16E-02	0.48E-01	GPI transamidase subunit PIG-U
IPR016088	7	4	0.96E-03	0.32E-01	Chalcone isomerase, 3-layer sandwich
IPR016363	4	4	3.30E-05	0.36E-02	Legume lectin
IPR017989	18	17	2.20E-16	5.17E-14	Ribosome-inactivating protein type 1/2
IPR021113	10	5	0.45E-03	0.21E-01	Acyl-ACP-thioesterase, N-terminal

Note: One-sided Fisher's exact test was used to test for significance.

Supplementary Table 12. GO enrichment of special groups in LJ43.

GO	All genes	Special genes	*P*-value	FDR	Function
GO:0030598	100	27	4.03E-09	5.24E-07	Ribosome-inactivating protein
GO:0043531	436	52	0.81E-03	0.31E-01	NB-ARC
GO:0045430	7	4	0.96E-03	0.31E-01	Chalcone isomerase, 3-layer sandwich
GO:0046961	10	5	0.45E-03	0.30E-01	ATPase, V1 complex, subunit H

Note: One-sided Fisher's exact test was used to test for significance.

Supplementary Table 13. KEGG enrichment of special groups in LJ43.

KEGG	All Genes	Special Genes	*P*-value	FDR	Function
K01859	7	4	0.96E-03	0.04	chalcone isomerase
K14153	8	5	0.12E-03	0.98E-02	hydroxymethylpyrimidine kinase phosphomethylpyrimidine kinase thiamine-phosphate diphosphorylase
K14305	16	9	5.71E-07	9.77E-05	nuclear pore complex protein Nup43
K14595	6	4	0.44E-03	0.02	abscisate beta-glucosyltransferase

Note: One-sided Fisher's exact test was used to test for significance.

Supplementary Table 14. The SNP results.

	Number	ts/tv	ts/tv (1st ALT)
InDel (only hard filter)	34,850,045	-	-
SNP (only hard filter)	387,042,351	2.27	2.66
Multiallelic SNP with	52,270,113 (13.51%)	0.96	1.41
SNP with InDel	18,118,594 (4.68%)	2.07	2.51
SNP (gap = 5 bp, qual>=40, MAF>=0.01, dp (2.5%~97.5%), biallelic SNP)	218,870,098 (56.55%)	3.56	3.56

Note: '-' indicates missing data.

Supplementary Table 15. Counts of the variant types in all tea samples.

Type (alphabetical order)	Count	Ratio (%)
initiator_codon_variant	297	0.00
intergenic_region	188,323,429	89.37
intron_variant	20,506,072	9.73
missense_variant	995,686	0.47
non_canonical_start_codon	4	0.00
splice_acceptor_variant	7,777	0.00
splice_donor_variant	6,369	0.00
splice_region_variant	117,681	0.06
start_lost	2,517	0.00
stop_gained	33,580	0.02
stop_lost	3,155	0.00
stop_retained_variant	1,722	0.00
synonymous_variant	734,138	0.35
exon	1,756,690	0.834
intergenic	188,323,429	89.42
intron	20,410,690	9.69
splice_site_acceptor	7,777	0.004
splice_site_donor	6,369	0.003
splice_site_region	95,641	0.045

Supplementary Table 16. GO enrichment of the SweepFinder2 results for CSA.

GO	Number	*P*-value	FDR	Function
GO:0000027	2	1.58E-03	3.88E-02	Midasin
GO:0000166	40	2.40E-04	1.18E-02	Nucleotide-binding alpha-beta plait domain
GO:0003676	63	2.60E-04	1.18E-02	Ribonuclease H-like domain
GO:0004379	2	1.58E-03	3.88E-02	Myristoyl-CoA:protein N-myristoyltransferase, N-terminal
GO:0004672	96	3.91E-05	3.93E-03	Protein kinase domain
GO:0005488	38	1.63E-05	2.40E-03	Armadillo-type fold
GO:0005515	222	2.54E-07	7.47E-05	Ankyrin repeat
GO:0005524	178	1.02E-11	6.03E-09	Protein kinase domain
GO:0006468	96	4.01E-05	3.93E-03	Protein kinase domain
GO:0008270	72	2.49E-04	1.18E-02	Transcription factor TFIIB
GO:0009234	2	1.58E-03	3.88E-02	Menaquinone biosynthesis protein MenD
GO:0016020	67	1.89E-04	1.11E-02	Pyrophosphate-energized proton pump
GO:0016021	62	3.05E-04	1.28E-02	Cornichon
GO:0016301	14	5.58E-07	1.10E-04	Diacylglycerol kinase, catalytic domain
GO:0016491	55	1.60E-04	1.05E-02	Polyketide synthase, enoylreductase domain
GO:0016638	3	1.15E-03	3.88E-02	Pyridoxine 5'-phosphate oxidase, dimerisation, C-terminal
GO:0016887	18	3.46E-04	1.36E-02	ABC transporter-like
GO:0031491	2	1.58E-03	3.88E-02	ISWI, HAND domain
GO:0042626	10	8.43E-04	3.10E-02	ABC transporter type 1, transmembrane domain
GO:0043044	2	1.58E-03	3.88E-02	ISWI, HAND domain
GO:0048278	2	1.58E-03	3.88E-02	Exocyst complex component Sec10-like
GO:0050242	2	1.58E-03	3.88E-02	Pyruvate, phosphate dikinase
GO:0055085	44	7.86E-05	5.78E-03	Sugar transporter, conserved site
GO:0055114	95	6.76E-05	5.69E-03	Alcohol dehydrogenase, C-terminal

Note: One-sided Fisher's exact test was used to test for significance.

Supplementary Table 17. GO enrichment of the SweepFinder2 results for CSS.

GO	Number	*P*-value	FDR	Function
GO:0000166	31	1.06E-03	3.56E-02	P-type ATPase, A domain
GO:0000178	2	9.38E-04	3.35E-02	Exosome complex component RRP45
GO:0000287	16	4.96E-06	8.86E-04	Terpene synthase, metal-binding domain
GO:0003910	3	1.43E-03	4.51E-02	DNA ligase, ATP-dependent, N-terminal
GO:0004185	11	4.56E-05	4.07E-03	Peptidase S10, serine carboxypeptidase
GO:0004373	3	9.15E-04	3.35E-02	Bacterial/plant glycogen synthase
GO:0005488	27	9.33E-04	3.35E-02	Armadillo-type fold
GO:0005515	160	3.46E-04	1.69E-02	Ankyrin repeat
GO:0005524	129	2.56E-07	9.02E-05	Protein kinase domain
GO:0006281	10	7.89E-04	3.35E-02	XPG/Rad2 endonuclease
GO:0006508	28	2.18E-04	1.30E-02	Peptidase S8/S53 domain
GO:0010333	9	1.32E-05	1.76E-03	Terpene synthase, N-terminal domain
GO:0016491	47	4.39E-05	4.07E-03	Oxoglutarate/iron-dependent dioxygenase
GO:0016829	9	5.51E-05	4.22E-03	Terpene synthase, N-terminal domain
GO:0016844	4	2.43E-04	1.30E-02	Strictosidine synthase, conserved region
GO:0043531	29	9.30E-05	6.23E-03	NB-ARC
GO:0055114	86	3.37E-07	9.02E-05	Cytochrome P450, E-class, group I

Note: One-sided Fisher's exact test was used to test for significance.

Supplementary Table 18. The average expression of selected terpene genes in CSS.

Gene	Bud	Flower	Leaf	Stem	Root
Cha06g010010	0.407081	0.257628	0.0419781	0.120084	0.494778
Cha12g011300	14.1382	0.384967	2.49478	0.441107	0.414616
Cha02g011720	0	0	0	10.0337	1.63255
Cha05g007640	0.074889	0	0	0.15892	0
Cha05g009080*	5.54577	0.704239	0.091366	0.182698	0.666819
Cha05g006930	1.40924	1.57527	0	0.371166	0.380853
Cha05g004010*	34.2759	19.6657	159.187	70.1075	16.6851
Cha10g002440	8.16136	6.08036	22.9976	0.00750407	20.0126
Cha09g013870*	6.23429	10.8835	29.8996	0.0307483	6.57594
Cha04g000480	0.0467892	1.4549	0	0.0198882	0.238279
Cha12g007000	0.947587	0.45224	0.894742	0.460001	0.827391
Cha04g015950	10.9641	0.955113	0	0.494315	0.0972183
Cha06g005810*	60.7087	0.173015	0.112903	1.51996	1.70406
Cha02g011980	1.96537	4.41655	13.1417	35.7095	2.82229
Cha02g011990*	0.417747	1.39067	11.0507	0.0579967	1.25263

Note: The average gene expression in the tissue was calculated, while one-sided t-tests were used to identify significant differences. ‘*’ indicates that the gene expression in the bud or leaf was significantly higher than that in other tissues.

Supplementary Table 19. The average expression of NB-ARC genes in CSS.

Gene	Spring	Summer	Autumn	Winter
Cha01g003880	0.385611	0.396025	0.493984	0.675391
Cha08g007820	0.0720208	0	0.0810365	0.0179926
Cha10g000620	3.20988	2.88009	1.96424	1.90201
Cha08g015930	4.36668	3.83948	3.99813	4.42616
Cha03g002160	0.0299588	0.154957	0.0776982	0.0200274
Cha13g002970*	12.0024	7.37154	9.10494	12.3034
Cha10g010310	0.291862	0.442867	0.313353	0.112577
Cha03g004080*	3.68761	4.74686	2.65007	1.90488
Cha05g009270*	1.15157	1.00833	0.515501	0.291326
Cha05g009330*	4.21901	0.945205	2.58612	2.58561
Cha13g002030	0.437237	0.464759	0.835618	0.206354
Cha15g001990	0.0729581	1.01925	0.155762	0.0641214
Cha15g001960	9.69115	2.97109	4.58856	8.01155
Cha03g014860	0.214578	0.230928	0.237035	0.129093
Cha02g002600	0.137786	0.303307	0.132342	0.0669902
Cha15g007110	1.21677	0.608785	1.08043	1.13186
Cha01g020790	6.37278	3.40941	4.19738	6.20538
Cha10g002650	0.753061	1.92344	0.588219	0.385155
Cha07g006220	0.565678	0.62565	0.528498	0.0950264
Cha08g015500	0.0950757	0.0418575	0.189681	0.209651
Cha14g004380*	6.82429	3.48099	8.11467	4.44938
Cha05g012050	2.89219	1.9246	3.30116	2.86623
Cha10g006590	0.251886	0.0882413	0.0810854	0.0168054
Cha09g010050*	3.37949	1.30861	2.70701	1.95418
Cha02g004880	2.95852	2.46588	2.26903	3.20311
Cha13g001370*	0.0120879	0.0417264	0.0832469	0.111354
Cha02g007120	0.795715	1.33952	0.957988	0.476364
Cha13g002260	4.25625	4.1153	4.29495	4.67308
Cha05g013820	0.938097	6.38732	1.10065	0.615442

Note: The average gene expression per season was calculated, while a one-sided t-test was used to identify significant differences. '*' indicates that the gene expression in the summer was significantly lower than that in other seasons. One-sided Fisher's exact test was used to test for significance.

Supplementary Table 20. The highly heterozygous genes retained in tea.

GO	Number
Cytochrome P450	12
UDP-glucuronosyl/UDP-glucosyltransferase	13
Small auxin-up RNA	2
Terpene synthase	3
Cytokinin dehydrogenase	1
Multi-antimicrobial extrusion protein	5
NB-ARC	10
S-locus	10
AP2/ERF domain	3
Malic oxidoreductase	1
NAC domain	4
WD40	8

Supplementary Table 21. The results of the F4 test and F3 test.

Samples	Statistic	Standard error	*Z*-score
DBZ-1,HZ002;HSKC,CM-1	-0.000260712	5.76778e-05	-4.52014
CSA2,CSR;CSA,CM-1	0.00696553	3.23781e-05	215.131
CSA,CSA2;CSR,CM-1	-0.0004924	1.89843e-05	-25.9373
HZ100,HZ118;HZ122,CM-1	-0.00312965	6.77548e-05	-46.1909
HZ100,HZ122;HZ118,CM-1	-0.00241358	6.89268e-05 -	35.0166
CSA,CSS2;CSS,CM-1	-0.0129479	5.63863e-05	-229.628
CSA,CSS;CSS2,CM-1	-0.013012	5.56352e-05	-233.88
CSA,CSA2;CSR,CM-1	-0.0004924	2.18646e-05	-22.5204
CSA2,CSR;CSA,CM-1	0.00696553	3.85973e-05	180.467
HZ104,HZ114;HZ117,CM-1	0.00619503	5.12426e-05	120.896
CSA,CSR;CSA2,CM-1	0.00647313	3.82574e-05	169.199
CSA2,CSA;CSR,CM-1	0.0004924	2.18646e-05	22.5204
HZ104,HZ117;HZ114,CM-1	0.00858967	5.77297e-05	148.791
DBZ-1,HSKC;HZ002,CM-1	0.00255663	6.59185e-05	38.7847
HZ039,HZ074;HZ092,CM-1	-0.000293896	5.69659e-05	-5.15916
HZ021,YNLDP1;HZ050,CM-1	0.00881408	5.79118e-05	152.198
HZ021,HZ050;YNLDP1,CM-1	0.00694945	5.04036e-05	137.876
HZ114,HZ117;HZ104,CM-1	0.00239463	7.2061e-05	33.2306
CSA2;CSA,CSR	0.00865398	3.81241e-05	226.995
CSA;CSA2,CSR	0.00669717	4.09978e-05	163.354
CSR;CSA,CSA2	0.0179839	4.66329e-05	385.648
CSA;CSS,CSS2	0.023809	6.07489e-05	391.924
CSS2;CSA,CSS	0.00712921	4.19279e-05	170.035
CSS;CSA,CSS2	0.00476459	4.15159e-05	114.766

Note: CSA included HZ114, HZ119, and HZ104; CSR included NC, HZ084, HZ001, XYDCS, LBDCS, and HZ027; CSA2 included HZ118, HZ122, HZ100, HZ072, HZ123, and HZ117; CSS included HZ050, HZ021, QXDM-1, and QXDM-2; CSS2 included HZ016, HZ036, HZ008, and HZ041. A,B;C,D represents an F4 test, and A;B,C represents an F3 test.

Supplementary References

1. Healey, A., Furtado, A., Cooper, T. & Henry, R.J. Protocol: a simple method for extracting next-generation sequencing quality genomic DNA from recalcitrant plant species. *Plant Methods* **10**, 21 (2014).
2. Bolger, A.M., Lohse, M. & Usadel, B. Trimmomatic: a flexible trimmer for Illumina sequence data. *Bioinformatics* **30**, 2114-2120 (2014).
3. Hanson, L., McMahon, K.A., Johnson, M.A.T. & Bennett, M.D. First nuclear DNA C-values for another 25 angiosperm families. *Ann. Bot.* **88**, 851-858 (2001).
4. Huang, H., Tong, Y., Zhang, Q.J. & Gao, L.Z. Genome size variation among and within *Camellia* species by using flow cytometric analysis. *Plos One* **8**, e64981 (2013).
5. Loureiro, J., Rodriguez, E., Dolezel, J. & Santos, C. Flow cytometric and microscopic analysis of the effect of tannic acid on plant nuclei and estimation of DNA content. *Ann. Bot.* **98**, 515-527 (2006).
6. Chikhi, R. & Medvedev, P. Informed and automated k-mer size selection for genome assembly. *Bioinformatics* **30**, 31-37 (2014).
7. Li, H. & Durbin, R. Fast and accurate short read alignment with Burrows-Wheeler transform. *Bioinformatics* **25**, 1754-1760 (2009).
8. Warren, R.L. *et al.* LINKS: Scalable, alignment-free scaffolding of draft genomes with long reads. *Gigascience* **4**, 35 (2015).
9. Yeo, S., Coombe, L., Warren, R.L., Chu, J. & Birol, I. ARCS: scaffolding genome drafts with linked reads. *Bioinformatics* **34**, 725-731 (2018).
10. Servant, N. *et al.* HiC-Pro: an optimized and flexible pipeline for Hi-C data processing. *Genome Biol.* **16**, 259 (2015).
11. Burton, J.N. *et al.* Chromosome-scale scaffolding of de novo genome assemblies based on chromatin interactions. *Nat. Biotechnol.* **31**, 1119-1125 (2013).
12. Wu, T.D. & Watanabe, C.K. GMAP: a genomic mapping and alignment program for mRNA and EST sequences. *Bioinformatics* **21**, 1859-1875 (2005).
13. Xu, Z. & Wang, H. LTR_FINDER: an efficient tool for the prediction of full-length LTR retrotransposons. *Nucleic Acids Res.* **35**, W265-W268 (2007).
14. Edgar, R.C. MUSCLE: multiple sequence alignment with high accuracy and high throughput. *Nucleic Acids Res.* **32**, 1792-1797 (2004).
15. Ou, S.J. & Jiang, N. LTR_retriever: A highly accurate and sensitive program for identification of long terminal repeat retrotransposons. *Plant Physiol.* **176**, 1410-1422 (2018).
16. Ou, S.J., Chen, J.F. & Jiang, N. Assessing genome assembly quality using the LTR Assembly Index (LAI). *Nucleic Acids Res.* **46**, e126 (2018).
17. Rhind, N. *et al.* Comparative functional genomics of the fission yeasts. *Science* **332**, 930-936 (2011).
18. Stanke, M., Tzvetkova, A. & Morgenstern, B. AUGUSTUS at EGASP: using EST, protein and genomic alignments for improved gene prediction in the human genome. *Genome Biol.* **7 Suppl 1**, S11 (2006).
19. Majoros, W.H., Pertea, M. & Salzberg, S.L. TigrScan and GlimmerHMM: two open source ab initio eukaryotic gene-finders. *Bioinformatics* **20**, 2878-2879 (2004).

20. Kaul, S. *et al.* Analysis of the genome sequence of the flowering plant *Arabidopsis thaliana*. *Nature* **408**, 796-815 (2000).
21. Goff, S.A. *et al.* A draft sequence of the rice genome (*Oryza sativa* L. ssp. *japonica*). *Science* **296**, 92-100 (2002).
22. Denoeud, F. *et al.* The coffee genome provides insight into the convergent evolution of caffeine biosynthesis. *Science* **345**, 1181-1184 (2014).
23. Argout, X. *et al.* The genome of *Theobroma cacao*. *Nat. Genet.* **43**, 101-108 (2011).
24. Jaillon, O. *et al.* The grapevine genome sequence suggests ancestral hexaploidization in major angiosperm phyla. *Nature* **449**, 463-467 (2007).
25. She, R., Chu, J.S.C., Wang, K., Pei, J. & Chen, N.S. GenBlastA: enabling BLAST to identify homologous gene sequences. *Genome Res.* **19**, 143-149 (2009).
26. Birney, E., Clamp, M. & Durbin, R. GeneWise and genomewise. *Genome Res.* **14**, 988-995 (2004).
27. Kim, D. *et al.* TopHat2: accurate alignment of transcriptomes in the presence of insertions, deletions and gene fusions. *Genome Biol.* **14**, R36 (2013).
28. Haas, B.J. *et al.* Automated eukaryotic gene structure annotation using EVidenceModeler and the program to assemble spliced alignments. *Genome Biol.* **9**, R7 (2008).
29. Li, R.Q. *et al.* SOAP2: an improved ultrafast tool for short read alignment. *Bioinformatics* **25**, 1966-1967 (2009).
30. Pertea, M., Kim, D., Pertea, G.M., Leek, J.T. & Salzberg, S.L. Transcript-level expression analysis of RNA-seq experiments with HISAT, StringTie and Ballgown. *Nat. Protoc.* **11**, 1650-1667 (2016).
31. Wei, C.L. *et al.* Draft genome sequence of *Camellia sinensis* var. *sinensis* provides insights into the evolution of the tea genome and tea quality. *Proc. Natl. Acad. Sci. USA* **115**, E4151-E4158 (2018).
32. Xia, E.H. *et al.* The tea tree genome provides insights into tea flavor and independent evolution of caffeine biosynthesis. *Mol. Plant* **10**, 866-877 (2017).
33. Zhu, C.Q. *et al.* Genome sequencing and CRISPR/Cas9 gene editing of an early flowering Mini-Citrus (*Fortunella hindsii*). *Plant Biotechnol. J.* **17**, 2199-2210 (2019).
34. Albert, V.A. *et al.* The *Amborella* genome and the evolution of flowering plants. *Science* **342**, 1241089 (2013).
35. Huang, S. *et al.* Draft genome of the kiwifruit *Actinidia chinensis*. *Nat. Commun.* **4**, 2640 (2013).
36. Guan, R. *et al.* Draft genome of the living fossil *Ginkgo biloba*. *Gigascience* **5**, 49 (2016).
37. Simao, F.A., Waterhouse, R.M., Ioannidis, P., Kriventseva, E.V. & Zdobnov, E.M. BUSCO: assessing genome assembly and annotation completeness with single-copy orthologs. *Bioinformatics* **31**, 3210-3212 (2015).
38. Jones, P. *et al.* InterProScan 5: genome-scale protein function classification. *Bioinformatics* **30**, 1236-1340 (2014).
39. Wang, Y.P. *et al.* MCScanX: a toolkit for detection and evolutionary analysis of gene synteny and collinearity. *Nucleic Acids Res.* **40**, e49 (2012).
40. Wang, W.S. *et al.* Genomic variation in 3,010 diverse accessions of Asian cultivated rice. *Nature* **557**, 43-49 (2018).

41. Tuskan, G.A. *et al.* The genome of black cottonwood, *Populus trichocarpa* (Torr. & Gray). *Science* **313**, 1596-1604 (2006).
42. Li, L., Stoeckert, C.J. & Roos, D.S. OrthoMCL: Identification of ortholog groups for eukaryotic genomes. *Genome Res.* **13**, 2178-2189 (2003).
43. Katoh, K. & Standley, D.M. MAFFT multiple sequence alignment software version 7: improvements in performance and usability. *Mol. Biol. Evol.* **30**, 772-780 (2013).
44. Stamatakis, A. RAxML version 8: a tool for phylogenetic analysis and post-analysis of large phylogenies. *Bioinformatics* **30**, 1312-1313 (2014).
45. Garcia-Andrade, J., Ramirez, V., Flors, V. & Vera, P. Arabidopsis *ocp3* mutant reveals a mechanism linking ABA and JA to pathogen-induced callose deposition. *Plant J.* **67**, 783-794 (2011).
46. Koh, E., Carmieli, R., Mor, A. & Fluhr, R. Singlet oxygen-induced membrane disruption and serpin-protease balance in vacuolar-driven cell death. *Plant Physiol.* **171**, 1616-1625 (2016).
47. Fourrier, N. *et al.* A role for *SENSITIVE TO FREEZING2* in protecting chloroplasts against freeze-induced damage in Arabidopsis. *Plant J.* **55**, 734-745 (2008).
48. Liu, J. & Last, R.L. MPH1 is a thylakoid membrane protein involved in protecting photosystem II from photodamage in land plants. *Plant Signal. Behav.* **10**, e1076602 (2015).
49. Yang, Z.H. PAML: a program package for phylogenetic analysis by maximum likelihood. *Comput. Appl. Biosci.* **13**, 555-556 (1997).
50. Lynch, M. & Conery, J.S. The evolutionary fate and consequences of duplicate genes. *Science* **290**, 1151-1155 (2000).
51. Schiffels, S. & Durbin, R. Inferring human population size and separation history from multiple genome sequences. *Nat. Genet.* **46**, 919-925 (2014).
52. Wang, G.D. *et al.* Out of southern East Asia: the natural history of domestic dogs across the world. *Cell Res.* **26**, 21-33 (2016).
