# Supplementary material for: Population sequencing enhances understanding of tea plant evolution
Source: Nat Commun. 2020 Sep 7;11:4447. doi: 10.1038/s41467-020-18228-8 (PMC7477583; doi:10.1038/s41467-020-18228-8)
Supplement: Supplementary file 4 — Description of Additional Supplementary Files [file 41467_2020_18228_MOESM4_ESM.pdf]

Description of Additional Supplementary Files

Supplementary Data 1
(InterPro) IPR enrichment of expansion genes.

Supplementary Data 2
The positive Darwinian selection genes of LJ43.

Supplementary Data 3
The information of tea population

Supplementary Data 4
The phylogenetic tree of all genome region with bootstrap. The phylogenetic tree was constructed by all genome SNPs.

Supplementary Data 5
The phylogenetic tree of inter gene region with bootstrap. We consider the selection status of SNPs, and the phylogenetic tree was constructed by inter gene region SNPs.

Supplementary Data 6
The phylogenetic tree of 4DTV. The phylogenetic tree was constructed by 4DTV sites.

Supplementary Data 7
The selection genes in CSS.

Supplementary Data 8
The selection genes in CSA.

Supplementary Data 9
The groups of the treemix.

Supplementary Data 10
F4-test of random individual.
