# Supplementary material for: Population sequencing enhances understanding of tea plant evolution
Source: Nat Commun. 2020 Sep 7;11:4447. doi: 10.1038/s41467-020-18228-8 (PMC7477583; doi:10.1038/s41467-020-18228-8)
Supplement: Supplementary file 9 — Supplementary Data 5 [file 41467_2020_18228_MOESM9_ESM.pdf]

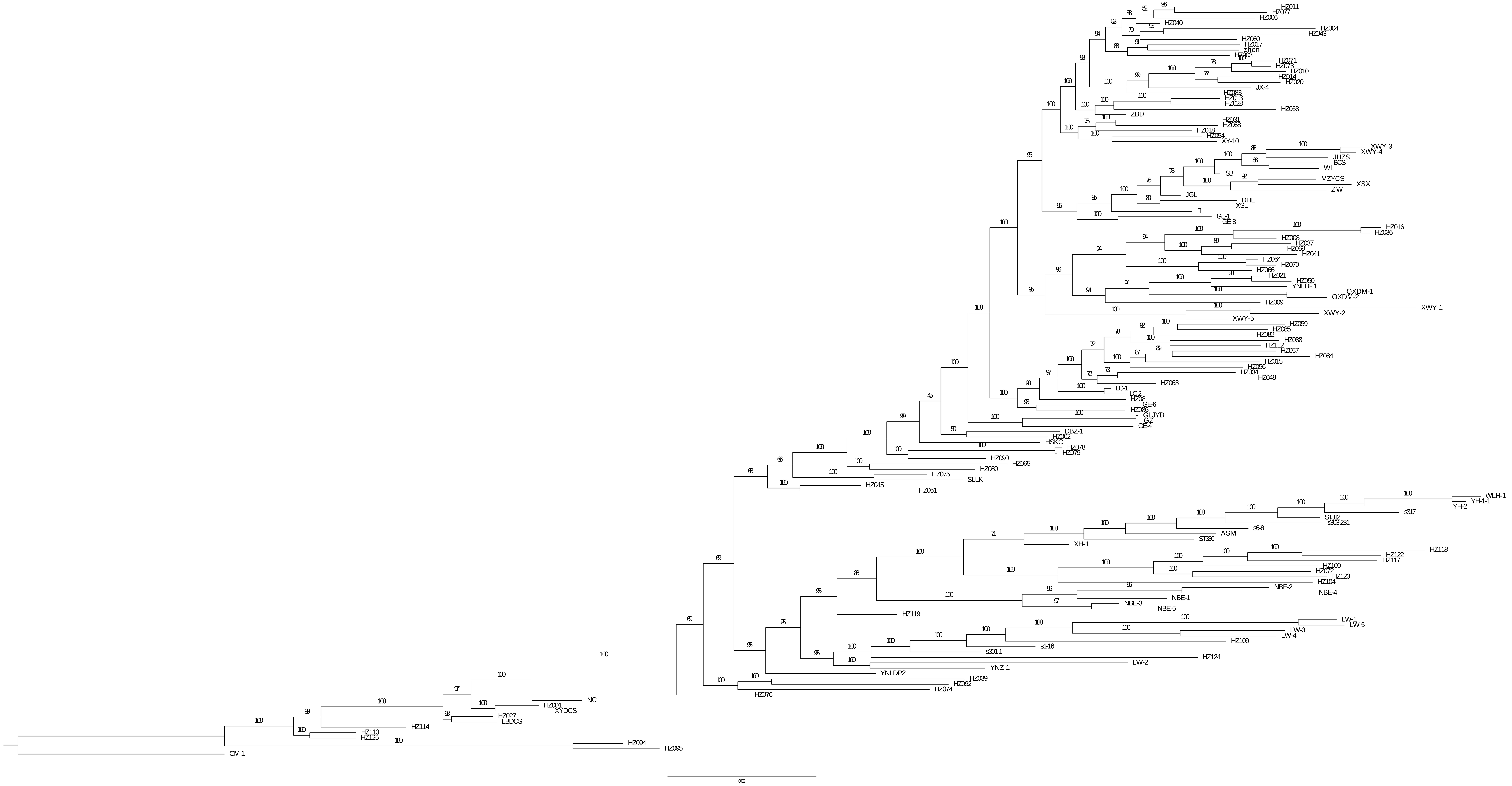
HZ011
HZ077
HZ006
HZ040
HZ004
HZ043
HZ060
HZ017
zhen
HZ003
HZ071
HZ073
HZ010
HZ014
HZ020
JX-4
HZ083
HZ013
HZ028
HZ058
ZBD
HZ031
HZ068
HZ018
HZ054
XY-10
XWY-3
XWY-4
JHZS
BCS
WL
SB
MZYCS
XSX
ZW
JGL
DHL
XSL
FL
GE-1
GE-8
HZ016
HZ036
HZ008
HZ037
HZ069
HZ041
HZ064
HZ070
HZ066
HZ021
HZ050
YNLDP1
QXDM-1
QXDM-2
HZ009
XWY-1
XWY-2
XWY-5
HZ059
HZ085
HZ082
HZ088
HZ112
HZ057
HZ084
HZ015
HZ056
HZ034
HZ048
HZ063
LC-1
LC-2
HZ081
GE-6
HZ086
GLJYD
GZ
GE-4
DBZ-1
HZ002
HSKC
HZ078
HZ079
HZ090
HZ065
HZ080
HZ075
SLLK
HZ045
HZ061
WLH-1
YH-1-1
YH-2
s317
ST312
s303-231
s6-8
ASM
ST330
XH-1
HZ118
HZ122
HZ117
HZ100
HZ072
HZ123
HZ104
NBE-2
NBE-4
NBE-1
NBE-3
NBE-5
HZ119
LW-1
LW-5
LW-3
LW-4
HZ109
s1-16
s301-1
HZ124
LW-2
YNZ-1
YNLDP2
HZ039
HZ092
HZ074
HZ076
NC
HZ001
XYDCS
HZ027
LBDCS
HZ114
HZ110
HZ125
HZ094
HZ095
CM-1
