# Supplementary material for: Population sequencing enhances understanding of tea plant evolution
Source: Nat Commun. 2020 Sep 7;11:4447. doi: 10.1038/s41467-020-18228-8 (PMC7477583; doi:10.1038/s41467-020-18228-8)
Supplement: Supplementary file 10 — Supplementary Data 6 [file 41467_2020_18228_MOESM10_ESM.pdf]

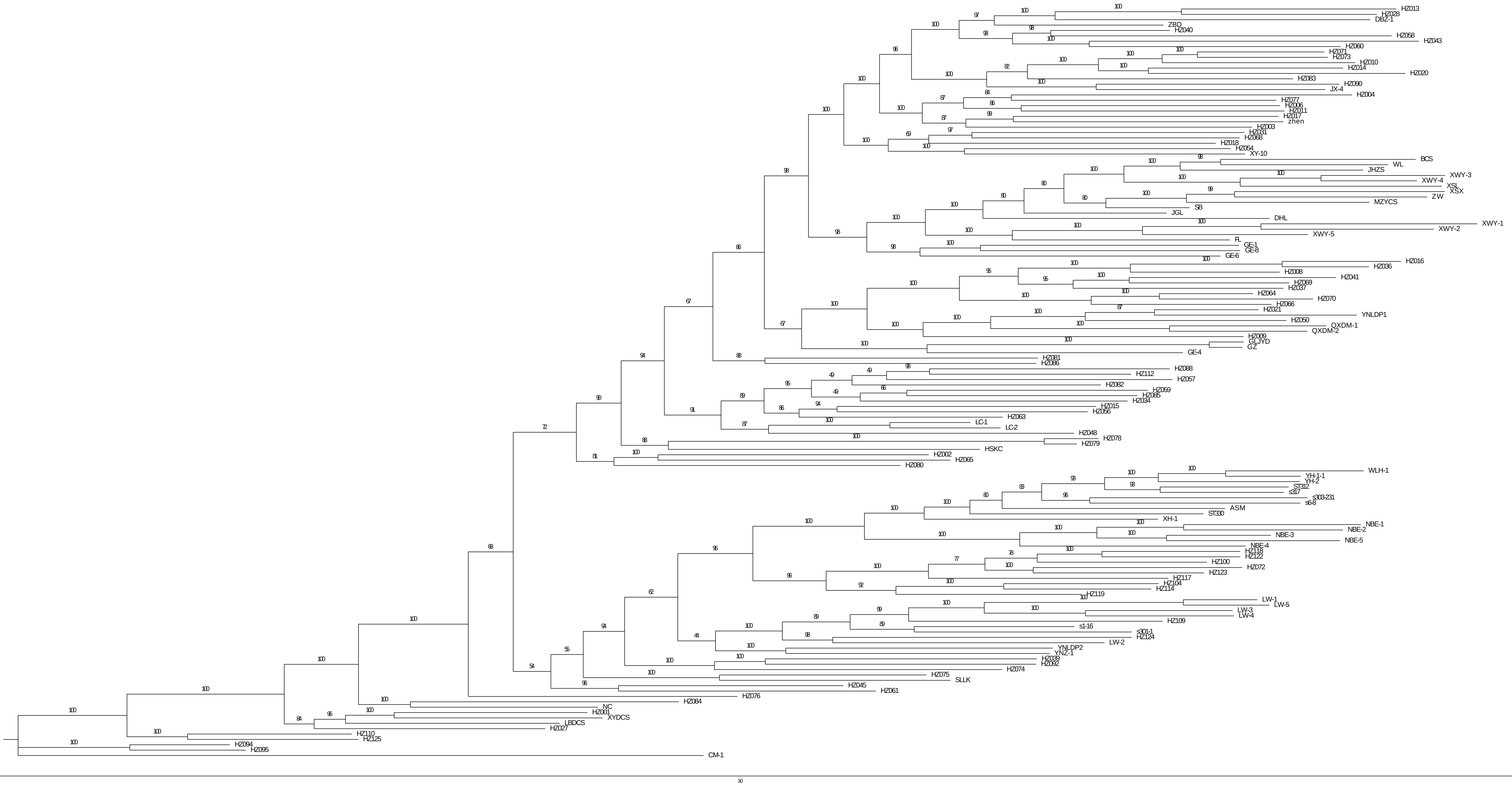
HZ013
HZ028
DBZ-1
ZBD
HZ040
HZ058
HZ043
HZ060
HZ071
HZ073
HZ010
HZ014
HZ020
HZ083
HZ090
JX-4
HZ004
HZ077
HZ006
HZ011
HZ017
zhen
HZ003
HZ031
HZ068
HZ018
HZ054
XY-10
BCS
WL
JHZS
XWY-3
XWY-4
XSL
XSX
ZW
MZYCS
SB
JGL
DHL
XWY-1
XWY-2
XWY-5
FL
GE-1
GE-8
GE-6
HZ016
HZ036
HZ008
HZ041
HZ069
HZ037
HZ064
HZ070
HZ066
HZ021
YNLDP1
HZ050
QXDM-1
QXDM-2
HZ009
GLJYD
GZ
GE-4
HZ081
HZ086
HZ088
HZ112
HZ057
HZ082
HZ059
HZ085
HZ034
HZ015
HZ056
HZ063
LC-1
LC-2
HZ048
HZ078
HZ079
HSKC
HZ002
HZ065
HZ080
WLH-1
YH-1-1
YH-2
ST312
s317
s303-231
s6-8
ASM
ST330
XH-1
NBE-1
NBE-2
NBE-3
NBE-5
NBE-4
HZ118
HZ122
HZ100
HZ072
HZ123
HZ117
HZ104
HZ114
HZ119
LW-1
LW-5
LW-3
LW-4
HZ109
s1-16
s301-1
HZ124
LW-2
YNLDP2
YNZ-1
HZ039
HZ092
HZ074
HZ075
SLLK
HZ045
HZ061
HZ076
HZ084
NC
HZ001
XYDCS
LBDCS
HZ027
HZ110
HZ125
HZ094
HZ095
CM-1
30
